# Augmentation of Saporin-Based Immunotoxins for Human Leukaemia and Lymphoma Cells by Triterpenoid Saponins: The Modifying Effects of Small Molecule Pharmacological Agents

**DOI:** 10.3390/toxins11020127

**Published:** 2019-02-20

**Authors:** Wendy S. Smith, David A. Johnston, Suzanne E. Holmes, Harrison J. Wensley, Sopsamorn U. Flavell, David J. Flavell

**Affiliations:** 1 The Simon Flavell Leukaemia Research Laboratory, Southampton General Hospital, Southampton SO16 6YD, UK; SuzanneH@leukaemiabusters.org.uk (S.E.H.); HarrisonW@leukaemiabusters.org.uk (H.J.W.); BeeF@leukaemiabusters.org.uk (S.U.F.); 2 Biomedical Imaging Unit, University of Southampton School of Medicine, Southampton General Hospital, Southampton SO16 6YD, UK; D.A.Johnston@soton.ac.uk

**Keywords:** saporin, immunotoxin, augmentation, saponin, endocytosis, reactive oxygen species

## Abstract

Triterpenoid saponins from *Saponinum album* (SA) significantly augment the cytotoxicity of saporin-based immunotoxins but the mechanism of augmentation is not fully understood. We investigated the effects of six small molecule pharmacological agents, which interfere with endocytic and other processes, on SA-mediated augmentation of saporin and saporin-based immunotoxins (ITs) directed against CD7, CD19, CD22 and CD38 on human lymphoma and leukaemia cell lines. Inhibition of clathrin-mediated endocytosis or endosomal acidification abolished the SA augmentation of saporin and of all four immunotoxins tested but the cytotoxicity of each IT or saporin alone was largely unaffected. The data support the hypothesis that endocytic processes are involved in the augmentative action of SA for saporin ITs targeted against a range of antigens expressed by leukaemia and lymphoma cells. In addition, the reactive oxygen species (ROS) scavenger tiron reduced the cytotoxicity of BU12-SAP and OKT10-SAP but had no effect on 4KB128-SAP or saporin cytotoxicity. Tiron also had no effect on SA-mediated augmentation of the saporin-based ITs or unconjugated saporin. These results suggest that ROS are not involved in the augmentation of saporin ITs and that ROS induction is target antigen-dependent and not directly due to the cytotoxic action of the toxin moiety.

## 1. Introduction

Immunotoxins (ITs) show high target cell specificity and cytotoxic potency making them potentially valuable candidate molecules for use as anti-tumour therapeutic agents. However, their clinical development has been hampered due to dose-limiting toxicities such as vascular leak syndrome and hepatotoxicity [1]. Improving the therapeutic index of ITs by reducing the dose at which a therapeutic response is achieved would represent a major advance for their use in cancer treatment. Several methods have been used previously to augment the toxicity of ITs including the use of virally derived peptides [2] and carboxylic ionophores [3]. Saporin is a 30kD proteinaceous ribosome inactivating protein derived from the plant *Saponaria officinalis*. Saporins mode of cytotoxicity for eukaryotic cells is to irreversibly inhibit protein synthesis via the catalytic removal of an adenine residue from 28S rRNA thereby irreversibly disrupting protein translation on the ribosome [4]. Possibly one of the most promising methods for saporin-based targeted toxin augmentation was first described by Heisler et al. [5] who demonstrated that saponins from Gypsophila plant species increased the cytotoxicity of epidermal growth factor (EGF)-saporin for the EGF^+^ human breast adenocarcinoma cell line MCF-7 and EGF transfected NIH-3T3 cells by several thousand-fold. This initial finding was subsequently extended to human leukaemia and lymphoma cell lines by Holmes et al. [6]. They investigated the effects of *Saponinum album* (SA) saponins on five saporin-based ITs, each against a different target molecule, and reported that the degree of augmentation varied considerably depending on the cell line and target molecule used. The membrane-lytic properties of saponins are well described and models such as pore formation [7], membrane vesiculation [8] and membrane lipid domain disruption [9] have been proposed to explain the perturbation of eukaryotic cell membranes by saponins. However, a sub-lytic concentration of SA possesses augmentative activity for IT cytotoxicity indicating that the mechanism of action probably does not involve plasma membrane permeabilisation [10]. The precise mechanism of saponin-mediated augmentation of targeted toxins is not yet fully characterized. SA augments the cytotoxicity of non-targeted unconjugated saporin (SAP) and also saporin that has been conjugated to both on and off-target antibodies as an IT [6]. This suggests that the augmentative effect is not dependent upon internalisation of the toxin via any single endocytic pathway. Saporin has been shown to specifically bind to the α_2_-macroglobulin receptor expressed by a wide variety of cell types and this would provide one potential route for receptor mediated endocytosis (RME) of the native toxin into the cell [11]. There is some limited experimental evidence to suggest that saporin is putatively internalised by clathrin-dependent RME into the endolysosomal system [12], though this remains to be independently confirmed. *Saponaria Officinalis* L. derived saponins also appear to modulate the release of saporin into the cytosol [13]. Therefore, a favoured hypothesis is that saponins cause the release of already internalised molecules from an intracellular vesicular compartment into the cytosol. It is currently not known whether saponins are internalised via an endocytic process from the fluid phase or, alternatively having bound to cholesterol in the plasma membrane, when sections of the plasma membrane are subsequently endocytosed. There may also be non-specific uptake of SA from the extra cellular fluid by macropinocytosis or non-clathrin-dependent endocytosis.

Bachran et al. [14] first demonstrated that a targeted toxin consisting of saporin 3 and epidermal growth factor (SE) in combination with SA entered cells via clathrin and actin dependent endocytic pathways. However, SE toxicity alone was unaffected by clathrin or actin blocking. As cargo progresses through the endosomal system the luminal pH drops progressively from 7.4 in the clathrin coated pit to pH 6.5–5.5 in early/late endosomes finally to pH 4.5 in the terminal lysosome. Holmes et al. [6] speculated that at lower pH the non-covalent interaction between saponin and saporin formed complexes that resulted in a conformational change in the saponin molecule consequently rendering it lytic for the endolysosomal membrane. This proposed model would require SA and IT to be taken into a common endosomal vesicle in order for SA-saporin complexes to form and then exert their lytic activity.

A co-localisation study in ECV-304 cells by Gilabert-Oriel et al. [15] demonstrated that alexafluor (AF) labelled saporin-trastuzumab was enriched in acidic vesicles such as endosomes and lysosomes in the absence of saponins. After addition of saponin SO1861 at a non-toxic concentration the escape of saporin-trastuzumab out of the endosomes or lysosomes into the cytosol was induced. The cell membrane was not affected, and the toxin remained inside the cell. Recent investigations in our laboratory have shown that endosomal release of SAP-AF was only clearly seen using SA at a concentration of 10 µg/mL after 15 h in Daudi cells (HJW unpublished observations). SA augmentation of saporin IT occurs using a concentration of 1 µg/mL SA. Therefore, the augmentative effect of SA on IT cytotoxicity might be dependent on other mechanisms in addition to increased endosomal escape. There may be later lysosomal membrane disruption by SA-saporin complexes resulting in the release of proteolytic enzymes that induce necrotic and apoptotic cell death once they have gained entry into the cytosol. Therefore, SA augmentation of IT cytotoxicity could involve several individual mechanisms.

Here we have investigated the effects of six inhibitory agents, known to affect clathrin-mediated endocytosis, endosomal acidification, actin polymerisation, macropinocytosis or microtubule formation, on SA augmentation of saporin-based ITs to gain further insight into the mechanism(s) of action. In addition, we have also determined the effect of tiron, a superoxide dismutase mimetic, on IT cytotoxicity alone or in combination with SA.

## 2. Results

The cytotoxicity of saporin ITs, alone and in combination with SA, towards Daudi, Ramos and HSB-2 cells has been previously described [6]. Here we investigated the effects of a range of small molecule pharmacological agents on the cytotoxicity of saporin ITs in the presence and absence of SA in Daudi and HSB-2 cells. The toxicity profiles of each compound for both cell lines were determined in order to select an appropriate sub-toxic concentration to be used in subsequent SA/IT augmentation experiments. The concentrations selected for use against Daudi cells were 100 µM chloroquine, 7.5 µM chlorpromazine, 0.75 µM cytochalasin D, 5 nM bafilomycin A1, 10 nM nocodazole and 25 µM ethylisopropylamiloride (EIPA) and the concentration curves are shown in Figure S1. The concentrations of the small molecule pharmaceutical agents used against HSB-2 cells were 10 µM chloroquine, 7.5 µM chlorpromazine, 5 nM bafilomycin A1, 0.75 µM cytochalasin D, 10 nM nocodazole and 20 µM EIPA (Figure S2).

**Figure 1:**
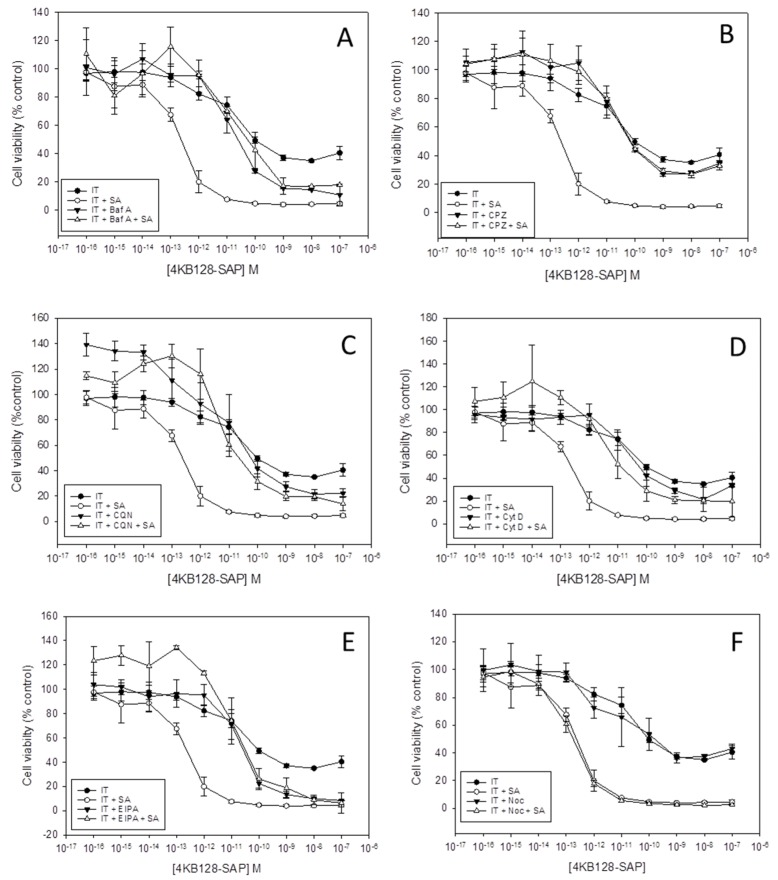
The effects of pharmacological agents on SA augmentation of 4KB128-SAP cytotoxicity in Daudi cells. Dose-response curves obtained by the XTT cytotoxicity assay for Daudi cells, in the absence (●) or presence (○) of 1 µg/mL SA, exposed to increasing concentrations of 4KB128-SAP compared to cells incubated with bafilomycin A1 (Baf A) (**A**), chlorpromazine (CPZ) (**B**), chloroquine (CQN) (**C**), cytochalasin D (Cyt D) (**D**), EIPA (**E**) or nocodazole (Noc) (**F**) in the absence (▼) or presence (∆) of 1 µg/mL SA and exposed to increasing concentrations of 4KB128-SAP. Samples were blank corrected and the absorbance at 470–650 nm was calculated for each well. Results are expressed as a percentage of control cells cultured in the relevant medium alone. Error bars represent the standard deviation either side of the mean for quadruplicate cultures and the data are representative of three independent experiments. Differences between the curves obtained for cells with IT plus SA without inhibitor and cells treated in the same way but with Baf A, Cpz, Cqn, Cyt D and EIPA were significant at the *p* < 0.01 level.

**Figure 2:**
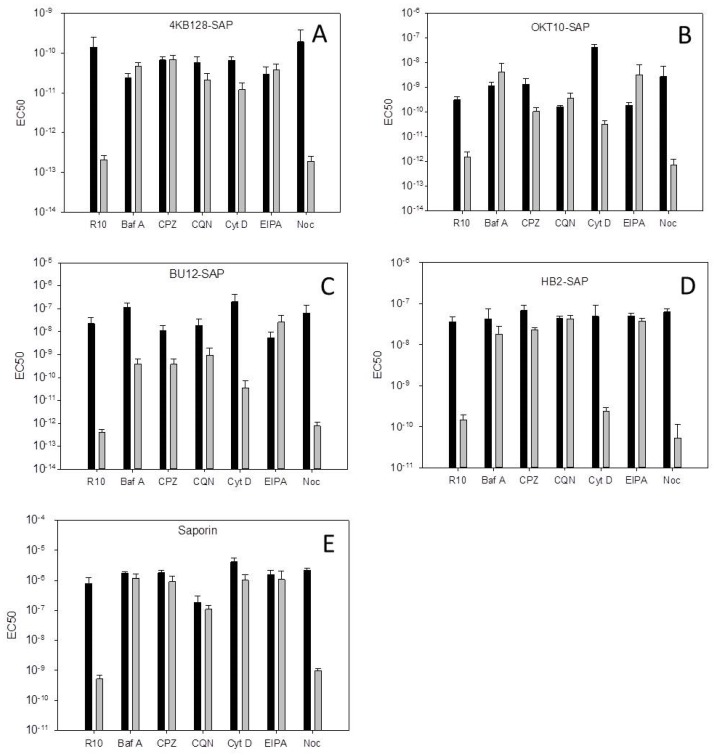
EC_50_ values for 4KB128-SAP (**A**), OKT10-SAP (**B**), BU12-SAP (**C**), HB2-SAP (**D**) and saporin (**E**) in Daudi cells treated with bafilomycin A1 (Baf A), chlorpromazine (CPZ), chloroquine (CQN), cytochalasin D (Cyt D), EIPA or nocodazole (Noc) calculated from the dose-response curves in Figure 1, Figures S4–S7 in the absence (black) or presence (grey) of SA. Error bars represent one standard deviation (SD) either side of the mean for three EC_50_ values.

Figure 1 presents the dose-response curves, as determined by the XTT (2,3-bis-(2-methoxy-4-nitro-5-sulphophenyl) 2H-tetrazolium-5-carboxanilide) assay, obtained for the Daudi cell line with the 4KB128-SAP (anti-CD22) IT in the presence of each pharmacological agent. The EC_50_ values determined for each IT or saporin tested against Daudi cells are presented in Figure 2 and the EC_50_ without SA/EC_50_ with SA ratios are shown in Table 1.

The EC_50_ values obtained from the dose-response curves using HSB-2 cells are presented in Table S1. Due to the lack of cytotoxicity of BU12-SAP and 4KB128-SAP towards off-target CD19 and CD22 negative HSB-2 cells it was not possible to determine EC_50_ values for these ITs alone.

SA alone at 1 µg/mL is not toxic towards Daudi and HSB-2 cells [6] and in our experiments did not become toxic when incubated with the inhibitors at the selected concentrations.

### 2.1. Inhibition of Clathrin-Mediated Endocytosis Abolishes SA Augmentation of Saporin Immunotoxins

Chlorpromazine is a cationic amphipathic drug that prevents the assembly and disassembly of clathrin lattices on the cell surface and on endosomes leading to inhibition of clathrin-mediated endocytosis (CME) [16] and has been widely used as an inhibitor of CME [14,17]. The cytotoxicity of the 4KB128-SAP IT alone was not affected by chlorpromazine (Figure 1B). However, when used in combination with SA, chlorpromazine reduced the cytotoxicity of the IT with SA from an EC_50_ of 2.1 × 10^−13^ M without chlorpromazine to 6.8 × 10^−11^ M with chlorpromazine (*p* < 0.01 as determined by Student’s *t* test). Similar effects on IT augmentation by SA were observed using the ITs OKT10-SAP (anti-CD38), BU12-SAP (anti-CD19), the off-target IT HB2-SAP (anti-CD7) and unconjugated saporin (Figures S4–S7).

Chlorpromazine completely abrogated the augmentative effect of SA for the on-target IT OKT10-SAP in HSB-2 cells (Figure S3B). This was also the case for the other three ITs and for unconjugated saporin. (Figures S8–S11 and Table S1).

### 2.2. Inhibition of Endosomal Acidification Abrogates SA Augmentation of Saporin Immunotoxins

Bafilomycin A1 and chloroquine both prevent acidification of endolysosomal vesicles, each via a different mechanism. Bafilomycin A1 is a strong inhibitor of the vacuolar H^+^ ATPase which leads to a decrease in luminal proton content and a subsequent increase in endosomal and lysosomal pH [18]. Chloroquine on the other hand diffuses into the cell and sequesters protons by becoming protonated itself within the acidic interior of endo-lysosomes and this leads to a direct increase in lysosomal pH [19]. In the presence of either bafilomycin A1 or chloroquine the cytotoxicity of the 4KB128-SAP IT for Daudi cells was not affected but both reagents significantly reduced the augmentative effect of SA for this IT (Figure 1A,C, respectively). The EC_50_ without SA/EC_50_ with SA decreased from 625 in cells exposed to IT plus SA without inhibitor to 0.5 (*p* < 0.05) and 2.7 (*p* < 0.05) in the presence of bafilomycin A1 and chloroquine respectively (Table 1). The augmentative effect of SA for on-target OKT10-SAP and BU12-SAP (anti-CD19), and the off-target IT HB2-SAP (anti-CD7) together with unconjugated saporin was also abrogated in Daudi cells by chloroquine and bafilomycin A1 (Figures S4–S7).

We also investigated the effects of bafilomycin A1 and chloroquine on SA augmentation of ITs in HSB-2 cells. Both compounds abrogated SA augmentation of the on-target OKT10-SAP (Figure S3) and HB2-SAP ITs (Figure S9), the off-target 4KB128-SAP IT (Figure S8) and unconjugated saporin (Figure S10). Only partial abrogation of SA augmentation by bafilomycin A1 was observed with BU12-SAP (Figure S11) compared to the complete abrogation observed after incubation with chloroquine.

### 2.3. Cytochalasin D Decreases OKT10-SAP Cytotoxicity

Cytochalasin D inhibits actin polymerisation causing disruption of actin microfilaments and activating p53 dependent pathways that lead to cell cycle arrest [20]. Cytochalasin D decreased the cytotoxicity of the OKT10-SAP IT for Daudi cells, increasing the EC_50_ from 3.1 × 10^−10^ M in the absence to 4 × 10^−8^ M in the presence of cytochalasin D (*p* = 0.01) as shown in Figure 2B and Figure S3D. In the presence of SA the EC_50_ of OKT10-SAP without cytochalasin D was 1.5 × 10^−12^ M but with cytochalasin D this increased to 3.2 × 10^−11^ M (*p* < 0.05). The EC_50_ “without SA/with SA” values were 240 and 1300 in control and cytochalasin D treated cells respectively (*p* < 0.01). The cytotoxic effects of 4KB128-SAP (Figure 1), HB2-SAP (Figure S7) and BU12-SAP (Figure S5) alone were unaffected by cytochalasin D. The presence of cytochalasin D led to partial abrogation of the augmentative effects of SA for BU12-SAP (Figure 2), and almost complete abrogation of SA augmentation for 4KB128-SAP (Figure 1D) and unconjugated saporin in Daudi cells (Figure 2).

Cytochalasin D also decreased the cytotoxic effect of OKT10-SAP IT in HSB-2 cells, reducing the EC_50_ from 5.2 × 10^−10^ M in control cells to 1 × 10^−7^ M in cytochalasin D treated cells (Figure S3D). The effect of cytochalasin D was more consistent in the HSB-2 cell line leading to almost complete abrogation of SA augmentation for all the ITs investigated together with unconjugated saporin (Figures S8–S11).

### 2.4. EIPA, An Inhibitor of Macropinocytosis, Abrogates SA Augmentation

The amiloride analogue EIPA has been widely used as an inhibitor of macropinocytosis [21,22] and acts by reducing the pH of the cytosolic face of the plasma membrane and preventing Rac1 and cdc42 signalling [23]. Figure 1E shows that a concentration of 25 µM EIPA abrogated the augmentative effect of SA for 4KB128-SAP IT cytotoxicity in Daudi cells. The EC_50_ “with SA/without SA” ratio decreased from 625 to 0.8 in the presence of EIPA (*p* < 0.05). Similar effects were obtained when Daudi cells were exposed to EIPA for on-target OKT10-SAP and BU12-SAP, off-target HB2-SAP and unconjugated saporin (Figures S4–S7). EIPA had no significant effect on IT cytotoxicity alone (Figure 1E and Figures S4–S7).

EIPA almost completely abrogated the augmentative effect of SA towards OKT10-SAP in HSB-2 cells but had no effect on OKT10-SAP cytotoxicity alone (Figure S3E). Similar results were obtained using the other three ITs investigated together with unconjugated saporin as shown in Figures S8–S11.

### 2.5. The Microtubule Disrupting Agent Nocodazole has No Effect on SA Augmentation of Saporin Immunotoxins

Nocodazole is a microtubule disrupting agent [24] that causes disturbances in microtubule dynamics which can lead to lysosomal instability [25]. Figure 1F shows that 10 nM nocodazole had no effect on the augmentative effect of SA towards 4KB128-SAP IT in Daudi cells. The augmentative effect of SA towards the ITs OKT10-SAP, BU12-SAP, HB2-SAP and unconjugated saporin was also unaffected (Figure S3). Nocodazole had no effect on IT cytotoxicity alone (Figure 1F and Figures S4–S7).

Nocodazole (10 nM) similarly had no effect on SA augmentation of OKT10-SAP in HSB-2 cells nor on OKT10-SAP cytotoxicity alone (Figure S3F). Figures S8–S11 show that there was no significant reduction in the SA augmentative effect towards 4KB128-SAP, BU12-SAP, HB2-SAP or saporin in the HSB-2 cell line.

### 2.6. Inhibitors of Endosomal Acidification Increase the Expression Level of CD22 in Daudi Cells

**Figure 3:**
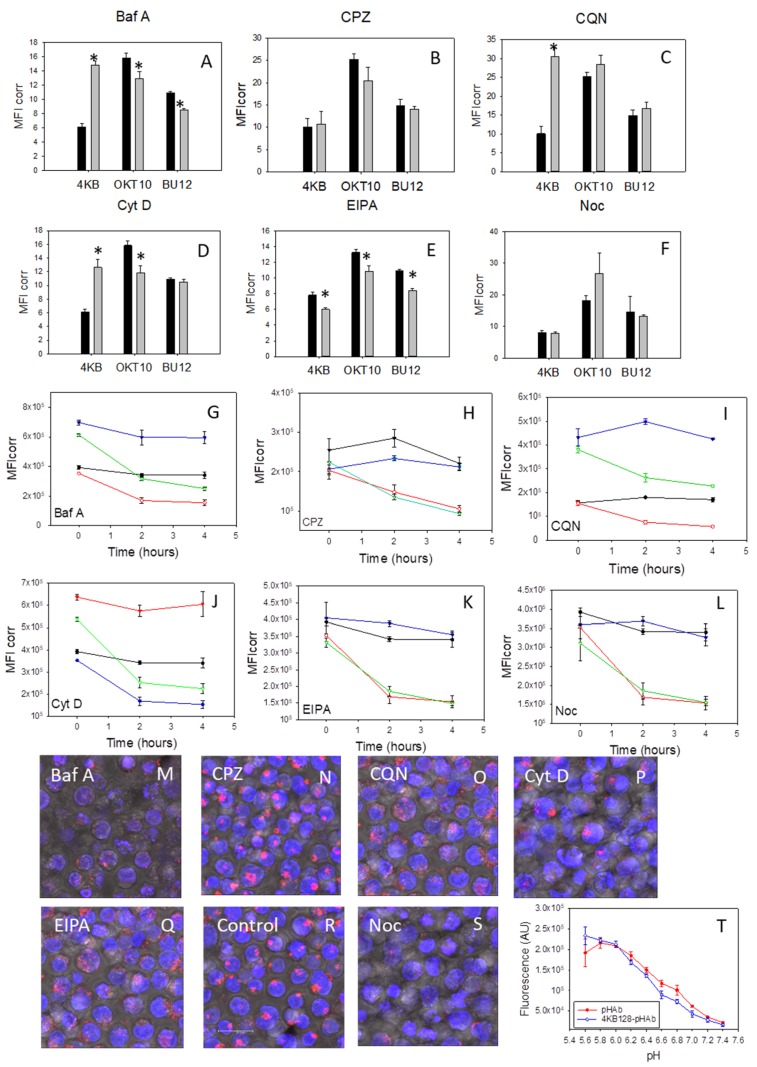
Inhibitors of endosomal acidification increase CD22 expression in Daudi cells. 4KB128, OKT10 and BU12 binding to Daudi control cells (black) or cells exposed to (**A**) bafilomycin A1 (grey), (**B**) chlorpromazine (grey), (**C**) chloroquine (grey), (**D**) cytochalasin D (grey), (**E**) EIPA (grey) and (**F**) nocodazole (grey) for 48 h. Results are plotted as mean fluorescence intensity (MFI). Values are the average of three to five replicates and representative of at least two independent experiments. * represents the degree of statistical significance as *p* < 0.005 Student’s *t* test relative to control. Time course of 4KB128 internalisation in Daudi control cells or cells treated with bafilomycin A1 (**G**), chlorpromazine (**H**), chloroquine (**I**), cytochalasin D (**J**), EIPA (**K**) and nocodazole (**L**) for 48 h. Control cells at 4 °C (—), control cells at 37 °C (—), treated cells at 4 °C (—), treated cells at 37 °C (—). Results are plotted as mean fluorescence intensity. The values presented are the average of duplicate cultures. The internalisation of 4KB128-pHAb in Daudi cells after 48 h exposure to bafilomycin A1 (**M**), chlorpromazine (**N**), chloroquine (**O**), cytochalasin D (**P**), EIPA (**Q**) and nocodazole (**S**) compared to control cells (**R**). Images are from samples taken after 24 h incubation with 4KB128-pHAb and a total of 48 h incubation with inhibitor. Images are maximum projections of a series of 26 z-slices at 1 µm spacing and are representative of two independent experiments. Scale bar represents 20 µm. (**T**) shows the change in pHAb (●) and 4KB128-pHAb (○) fluorescence over the pH range 5.6 to 7.4. Values are the average of duplicate measurements.

In order to exclude the possibility that the abrogation of SA augmentation of IT cytotoxicity, by the inhibitors studied, was due to reduced target antigen expression levels and/or internalisation following antibody ligation we investigated the effects of six pharmacological agents on CD22 expression and its internalisation by Daudi cells by following cognate antibody binding to cell surface CD22 using flow cytometry. The results obtained are shown in Figure 3 and reveal a two-fold increase in the mean fluorescence intensity (MFI) value obtained for 4KB128 antibody binding in Daudi cells treated with bafilomycin A1 for 48 h (Figure 3A) compared to untreated control cells. Similarly, we observed a three-fold increase in 4KB128 antibody staining in cells treated with chloroquine (Figure 3C) for 48 h and a two-fold increase in the MFI value determined for Daudi cells exposed to cytochalasin D (Figure 3D). Nocodazole (Figure 3F) and chlorpromazine (Figure 3B) had no effect on the expression level of CD22 measured as 4KB128 antibody binding. EIPA produced a small but significant decrease in 4KB128 antibody binding (*p* < 0.005 Student’s *t* test with a 2 tailed distribution) in Daudi cells (Figure 3E). Bafilomycin A1 and EIPA reduced the level of apparent CD19 expression as determined by BU12 antibody binding. Treatment with nocodazole increased OKT10 antibody binding to Daudi cells (Figure 3F) indicating increased CD38 surface expression (*p* < 0.01). Bafilomycin A1, cytochalasin D and EIPA produced small but significant decreases in CD38 expression demonstrable as reduced OKT10 antibody binding (*p* < 0.005).

Figure 3G–L show that CD22 was lost from the cell surface at a similar rate in cells incubated with chlorpromazine (Figure 3H), chloroquine (Figure 3I), bafilomycin A1 (Figure 3G), cytochalasin D (Figure 3J), EIPA (Figure 3K), nocodazole (Figure 3L) and mock treated control cells even though the initial level of 4KB128 binding was elevated in chloroquine, bafilomycin A1 and cytochalasin D treated cells.

We next investigated the internalisation of pHAb labelled 4KB128 antibody (4KB128-pHAb) into treated and control cells using confocal microscopy. The pHAb dye does not fluoresce at neutral pH but becomes fluorescent at acidic pH. The fluorescence responses of pHAb and 4KB128-pHAb to pH are shown in Figure 3T. Therefore, when 4KB128-pHAb is internalised and trafficked into endolysosomal vesicles the luminal pH progressively decreases and the dye fluoresces [26]. Daudi cells incubated with chloroquine for 48 h produced a less punctate distribution of 4KB128-pHAb compared to control cells as measured after incubation with the labelled antibody for 24 h (Figure 3O,R). Bafilomycin A1 caused a reduction in the fluorescence intensity of 4KB128-pHAb compared to control cells but the distribution still remained punctate (Figure 3M). The distribution and intensity of 4KB128-pHAb in cells incubated with chlorpromazine (Figure 3N) was near identical to that exhibited by control cells (Figure 3R). Cytochalasin D treated Daudi cells appeared swollen (Figure 3P) and some cells had distorted nuclei with approximately 50% of the treated cells showing a similar punctate distribution of 4KB128-pHAb to that seen in control cells. The fluorescence due to 4KB128-pHAb in nocodazole treated cells was clearly reduced (Figure 3S) in comparison to that detected in untreated Daudi cells (Figure 3R). In contrast Daudi cells incubated with EIPA for 48 h produced a less punctate distribution of pHAb (Figure 3Q) to that seen in control cells (Figure 3R).

### 2.7. CD7 and CD38 Expression Levels in HSB-2 Cells Are Unaffected by Exposure to Inhibitors

**Figure 4:**
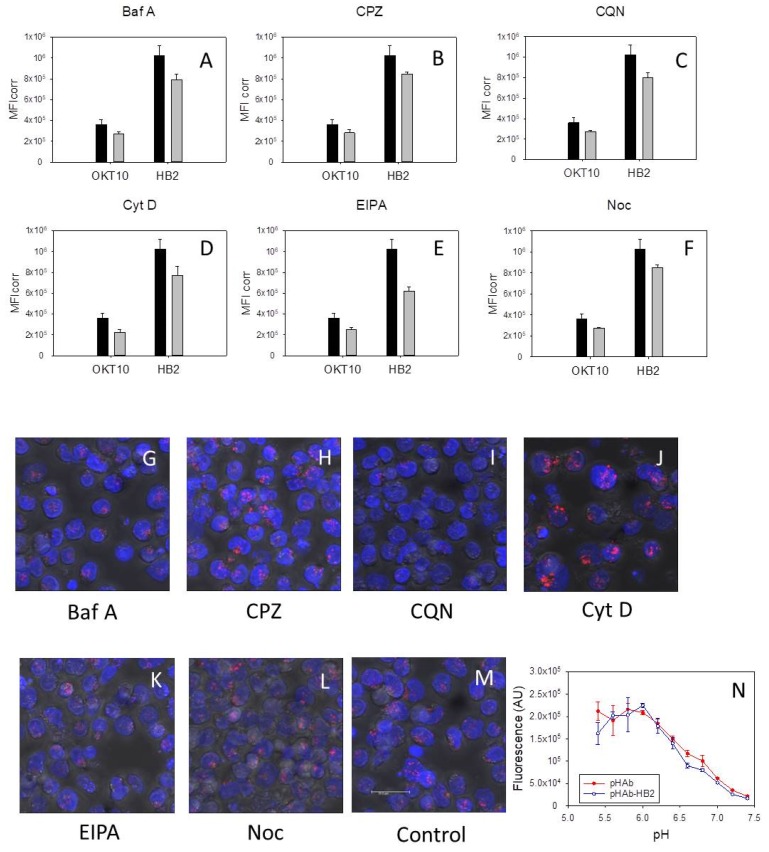
The effects of pharmacological agents on CD7 and CD38 expression and internalisation in HSB-2 cells. OKT10 and HB2 binding to HSB-2 control cells (black) or cells exposed to (**A**) bafilomycin A1 (grey), (**B**) chlorpromazine (grey), (**C**) chloroquine (grey), (**D**) cytochalasin D (grey), (**E**) EIPA (grey) and (**F**) nocodazole (grey) for 48 h. Results are plotted as mean fluorescence intensity (MFI). Values are the average of three replicates and representative of at least two independent experiments. Internalisation of HB2-pHAb in HSB-2 cells after 48 h exposure to bafilomycin A1 (**G**), chlorpromazine (**H**), chloroquine (**I**), cytochalasin D (**J**), EIPA (**K**) and nocodazole (**L**) compared to control cells (**M**). Images are from samples taken after 24 h incubation with pHAb-HB2 and a total of 48 h incubation with inhibitor. Images presented are maximum projections of a series of 21 z-slices at 1 µm spacing and are representative of two independent experiments. Scale bar represents 20 µm. Changes in pHAb (●) and HB2-pHAb (○) fluorescence with increasing pH (**N**). Values are the average of duplicate measurements.

Figure 4A–F show the apparent surface expression levels of CD38 and CD7 in untreated HSB-2 cells and HSB-2 cells after exposure to bafilomycin A1, chlorpromazine, chloroquine, cytochalasin D, EIPA or nocodazole for 48 h.

There were no major differences in OKT10 and HB2 binding between control and treated HSB-cells after exposure to bafilomycin A1 (Figure 4A), chlorpromazine (Figure 4B), chloroquine (Figure 4C), cytochalasin D (Figure 4D), EIPA (Figure 4E) or nocodazole (Figure 4F). We also investigated the internalisation of HB2-pHAb into treated and control cells using confocal microscopy. Figure 4N shows the fluorescence of the HB2-pHAb conjugate and the pHAb label alone over the pH range pH 5.4 to pH 7.4. HSB-2 cells incubated with chlorpromazine for 48 h showed a similar intensity and distribution of HB2-pHAb to that produced in control cells (Figure 4H,M, respectively). HSB-2 cells exposed to cytochalasin D were enlarged and the fluorescence due to HB2-pHAb appeared more intense (Figure 4J). HSB-2 cells exposed to chloroquine showed reduced HB2-pHAb fluorescence although the nuclei of some cells were distorted (Figure 4I). The distribution of HB2-pHAb in bafilomycin A1 treated cells still remained localised but was slightly less intense (Figure 4G) than that seen in control cells. In EIPA and nocodazole treated cells (Figure 4K,L, respectively) the distribution and intensity of HB2-pHAb was similar to that seen in control cells.

### 2.8. Cell Cycle Analysis

**Figure 5:**
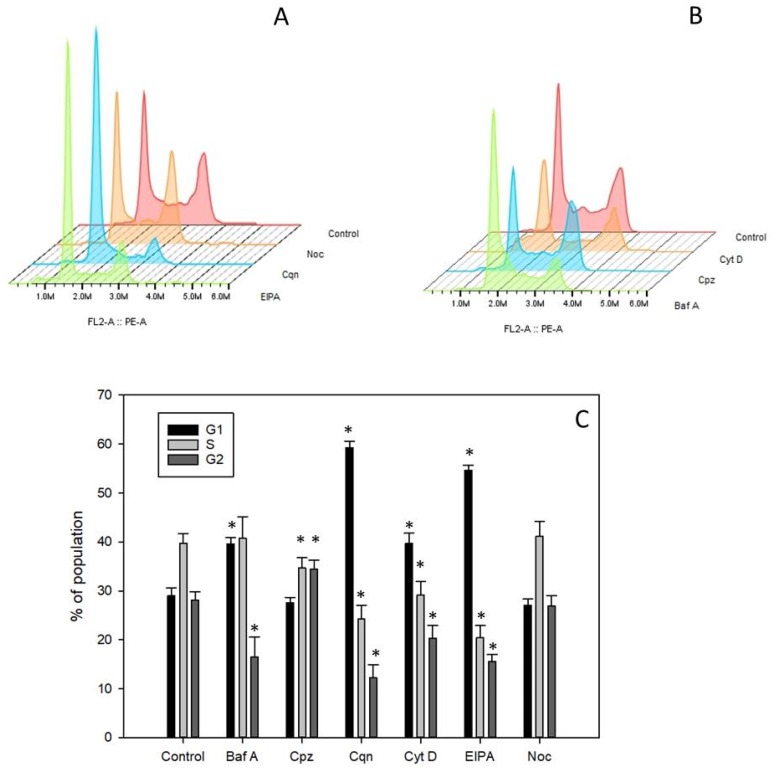
Cell cycle analysis of Daudi cells exposed to pharmacological agents. (**A**) Cell cycle profiles of Daudi cells treated with nocodazole (Noc), chloroquine (Cqn) or EIPA compared to untreated control cells. (**B**) Cell cycle profiles of Daudi cells treated with cytochalasin D (Cyt D), chlorpromazine (Cpz) or bafilomycin A1 (Baf A) compared to untreated control cells. (**C**) Relative percentages of cells in G1, S and G2 as determined using the Dean-Jett-Fox algorithm and Flow Jo v 10.5.2. Values are the average of five replicates and the data are representative of two independent experiments. * represents statistical significance relative to control at the *p* < 0.005 level by Student’s *t* test.

We examined the cell cycle distribution of Daudi cells exposed to the six small molecule pharmacological agents under investigation. Untreated Daudi control cells displayed a cell cycle distribution of approximately 29% in G_1_, 40% in S and 28% in G_2_M (Figure 5A–C).

Daudi cells incubated with chloroquine for 48 h accumulated in G_1_ (~59%) with the number of cells in the S and G_2_M phases both being concomitantly reduced (Figure 5A,C). Bafilomycin A1 led to an increase in the percentage of cells in G_1_, up to 40%, and a decrease in G_2_M (Figure 5B,C). Cytochalasin D caused an increase in the G_1_ population (up to 40%) and a decrease in S and G_2_M (Figure 5B,C). EIPA also produced an increase in the G_1_ population (up to 55%) with a concomitant decrease in G_2_M and S phases (Figure 5A,C). Chlorpromazine had no significant effect on the percentage of cells in G1 but a significantly higher percentage of cells accumulated in G2 with a concomitant decrease in S phase (Figure 5B,C). Cells treated with nocodazole produced a similar cell cycle distribution to that observed in control cells (Figure 5A,C).

### 2.9. Tiron, A Superoxide Scavenger, Reduces OKT10-SAP Cytotoxicity in Daudi Cells

**Figure 6:**
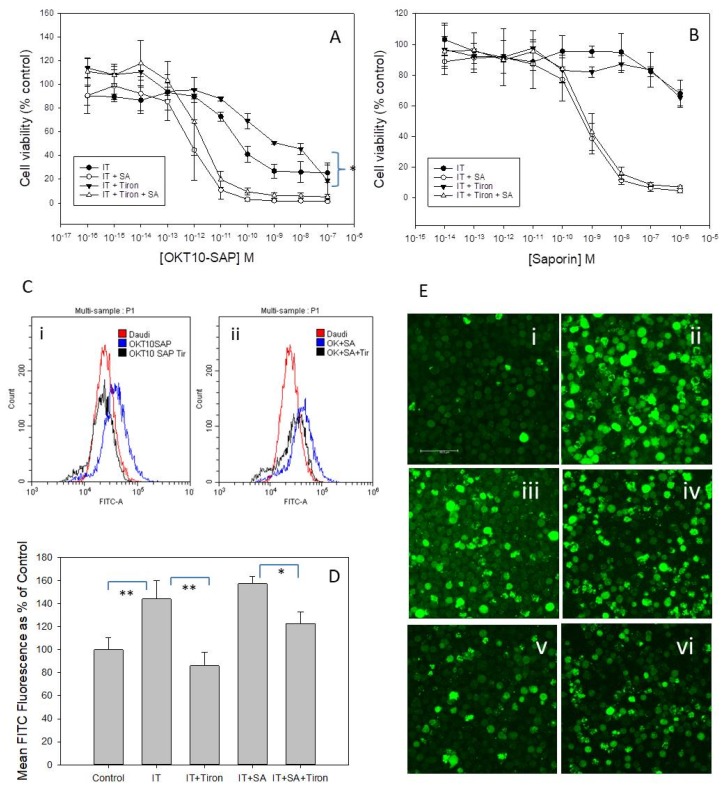
The effects of tiron on SA augmentation of OKT10-SAP or saporin in Daudi cells. Dose-response curves obtained by the XTT assay for mock treated Daudi cells in the absence (●) or presence (○) of 1 µg/mL SA, exposed to increasing concentrations of OKT10-SAP (**A**) or saporin (**B**) compared to cells incubated with 500 µM tiron (**A**) in the absence (▼) or presence (∆) of 1 µg/mL SA, following incubation in RPMI 1640 medium or 1 mM tiron for 1 h. Error bars represent the standard deviation either side of the mean for three independent experiments each with quadruplicate samples. The significance of differences between the curves for IT alone or IT plus SA were evaluated using two-way ANOVA. * *p* = 0.0137. (**C**)(**i**) Flow cytometric traces showing CellRox green fluorescence in Daudi cells exposed to OKT10 SAP (blue) or OKT10-SAP (1 × 10^−10^ M) plus tiron (black) for 48 h compared to mock treated control cells (red). (**ii**) Flow cytometric traces showing CellRox green fluorescence in Daudi cells exposed to OKT10 SAP (1 × 10^−12^ M) and SA (blue), OKT10-SAP and SA plus tiron (1 h pre-incubation with 1 mM followed by 500 nM for 48 h) (black) compared to mock treated control cells (red). (**D**) Mean FITC fluorescence of Daudi cells exposed to OKT10-SAP or OKT10-SAP plus SA in the presence of 500 nM tiron, expressed as a percentage of values obtained from mock treated Daudi control cells. Results are the average of two independent experiments and * represents *p* < 0.05, ** represents *p* < 0.01 Student’s *t* test. (**E**) Carboxy H_2_DCFDA fluorescence in Daudi control cells (**i**) and hydrogen peroxide treated cells (**iv**) compared to cells treated with OKT10-SAP (**ii**), OKT10-SAP plus 500 nM tiron (**iii**), OKT10-SAP with SA (**v**) and OKT10-SAP with SA plus tiron (**vi**) for 48 h. Images presented are maximum projections of a series of 26 z-slices at 1 µm spacing. Scale bar represents 60 µm.

We next investigated whether saporin ITs in the presence or absence of SA induced ROS to mediate cell death. We determined the effects of the superoxide scavenger tiron on OKT10-SAP cytotoxicity in Daudi cells in the presence and absence of 1 µg/mL SA. The dose-response curves obtained by the XTT assay for Daudi cells incubated with and without tiron for 48 h are presented in Figure 6A. Pre-incubation of Daudi cells for 1 h with 1 mM tiron prior to incubation for 48 h with 500 µM tiron produced a significant reduction in OKT10-SAP cytotoxicity (*p* = 0.0137). However, tiron did not significantly affect the cytotoxicity of OKT10-SAP and SA when used in combination (*p* = 0.135). Tiron had no demonstrable effect on saporin cytotoxicity alone (Figure 6B) nor on SA augmentation of saporin. The cytotoxicity of BU12-SAP IT used alone was also reduced by tiron but 4KB128-SAP IT cytotoxicity was unaffected (Figure S12).

We used CellRox Green to measure intracellular ROS levels by flow cytometry and showed that treatment with tiron reduced the level of ROS in Daudi cells exposed to OKT10-SAP or OKT10-SAP plus SA in comparison to cells exposed only to IT or IT plus SA without tiron. Individual flow cytometric traces (Figure 6C) showed an increased but broad distribution of ROS in cells treated with OKT10-SAP in combination with SA. In the presence of tiron the population remained broad in distribution but there was a decrease in the level of ROS detected. Figure 6D presents the mean fluorescein isothiocyanate (FITC) fluorescence as a percentage of control cell FITC fluorescence for Daudi cells treated with IT and IT plus SA with and without tiron. The dye 5-(and 6)-carboxy-2′, 7′-dichlorodihydrofluorescein diacetate (carboxy-H_2_DCFDA) was also used as a probe for ROS detection in live cell imaging studies. Carboxy-H_2_DCFDA is oxidized in the presence of ROS to carboxy-DCF which emits green fluorescence. Daudi cells exposed to IT (Figure 6E(ii)) or IT plus SA (Figure 6E(v)) showed increased carboxy-H_2_DCFDA fluorescence compared to control cells (Figure 6E(i,iv)). Daudi cells exposed to tiron prior to and during incubation with IT (Figure 6E(iii)) and IT plus SA (Figure 6E(vi)) showed reduced fluorescence compared to cells exposed only to IT (Figure 6E(ii)) or to IT in combination with SA (Figure 6E(iv)). Therefore, these imaging data support the results obtained using CellRox Green.

## 3. Discussion

The purpose of this study was to determine the effects of a range of small molecule agents, with established pharmacological activities, on the augmentative effect of SA for IT cytotoxicity in order to gain an insight into the mechanism(s) of augmentation. We have clearly shown that chlorpromazine, commonly used as an inhibitor of clathrin-mediated endocytosis (CME) [27], reduced the augmentative effect of SA for both on and off-target saporin ITs and unconjugated saporin in both Daudi and HSB-2 cells. This is in agreement with work reported by Bachran et al. [14] who showed that chlorpromazine significantly reduced the augmentative effect of SA on the cytotoxicity of the targeted toxin (TT) Saporin-EGF (SE) towards HER14 cells (a Swiss mouse embryo cell line stably transfected with human EGF receptor) when used in combination with SA. In our study the cytotoxicity of the IT alone was not altered by chlorpromazine indicating that IT access into an intracellular compartment is not clathrin dependent. We observed that there was no effect on the apparent expression levels of CD19, CD22 or CD38 in Daudi cells exposed to chlorpromazine and that CD22 appeared to be lost from the Daudi cell surface at a similar rate in the presence or absence of chlorpromazine. Uptake of 4KB128-pHAb (anti-CD22) was similar in control and chlorpromazine treated Daudi cells and there was no apparent effect on the cell cycle. We therefore conclude that CME is centrally involved in the SA augmentation of saporin ITs.

Chloroquine and bafilomycin A1 had no effect on the cytotoxicity of IT alone in both Daudi and HSB-2 cells indicating that access of the toxin to the cytosol from an intracellular vesicle is pH independent. In contrast the augmentative effect of SA was abrogated by both chloroquine and bafilomycin A1. These observations independently confirm the work of Bachran et al. [14] in a different tumour and target antigen system. These authors proposed that SA integrates into endosomal membranes and then mediates transfer of SE into the cytosol speculating that endosomal acidification is necessary for one or both of these steps. There is some tentative experimental evidence to suggest that saponins and ribosome inactivating proteins (RIPs) form non-covalent associations at an acidic pH as encountered in the lumen of the late endolysosomal system [28]). This may lead to the formation of a membrane-lytic complex that disrupts the vesicle membrane. Alternatively, saponin contained within an acidic vesicle lumen may simply cause direct disruption of the membrane.

The increased expression levels of CD22 we observed in cells treated with bafilomycin and chloroquine may be due to CD22 recycling since O’ Reilly et al. [29] showed that antibodies bound to CD22 do not dissociate following RME but are recycled back to the cell surface. Chloroquine and bafilomycin, A1 by preventing acidification, may prevent the dissociation and subsequent intracellular degradation of CD22 bound 4KB128-SAP following RME thus enabling greater amounts of CD22 to recycle back to the cell surface. We observed that the distribution of 4KB128-pHAb in chloroquine treated Daudi cells was less intense and less tightly distributed than that observed in control cells. There was also a slight reduction in the fluorescence intensity of HB2-pHAb in chloroquine treated HSB-2 cells. These decreases in intensity are probably due to a change in endolysosomal pH rather than a reduction in uptake of the antibody. The acidic pH in endocytic and exocytic compartments can be raised to neutrality by bafilomycin depending on the concentration applied [18,30]. If bafilomycin causes a greater shift in endosomal pH than chloroquine this would explain the lower fluorescence of 4KB128-pHAb in bafilomycin A1 treated Daudi cells compared to chloroquine treated Daudi cells. Variable abnormal pH regulation in the vesicular compartments of cancer cells could explain the variations that are reported in the literature on the augmentative effects of saponins on saporin-based targeted toxins for different cell lines [6,31].

Treatment of cells with agents that prevent actin dynamics hinders the assembly of clathrin coated pits and vesicles and the subsequent endocytosis of transferrin [32]. Cytochalasin D, used in our studies as an inhibitor of actin polymerisation, reduced the cytotoxicity of OKT10-SAP towards Daudi and HSB-2 cells. However, cytochalasin D did not reduce the cytotoxicity of the other three (anti-CD7, -CD19 and -CD22) saporin ITs investigated. This variation might indicate the differences that exist for each target molecule’s association with PM cholesterol and /or the actin membrane skeleton. In this context Jiang et al. [33] found that cytochalasin D significantly reduced the capability of the anti-CD38 antibody SAR650984 (SAR) to kill CD38^+^ multiple myeloma cells and greatly reduced SAR induced homotypic adhesion. Cytochalasin D abrogated SA augmentation of all of the ITs tested against HSB-2 cells. This contrasts with the variable, partially target molecule specific, effect of cytochalasin D observed in Daudi cells. Fujimoto et al. [34] reported that cytochalasin D exerted variable effects on RME in several different cell lines, concluding that actin assembly plays a variable but not obligatory role in RME.

Nocodazole acts on microtubules that are required for the normal positioning and movement of intracellular vesicular compartments [35]. Nocodazole does not affect endosomal pH but has been reported to inhibit endosomal transport from endosomal carrier vesicles (ECV) to late endosomes [35] arresting transfer from compartments with an average pH of 6.0 to compartments of pH 5.2 (i.e., lysosomes) [30]. In the present study we found that nocodazole had no effect on IT or saporin cytotoxicity or on the augmentative effect of SA for IT cytotoxicity in either Daudi or HSB-2 cells. There was a reduction in the fluorescence intensity of 4KB128-pHAb in Daudi cells but the fluorescence of HB2-pHAb was similar in control and nocodazole treated HSB-2 cells. Therefore, we suggest that in our experiments, nocodazole, might have caused the accumulation of 4KB128-pHAb in endocytic compartments of higher pH resulting in a reduced fluorescence signal from the pH sensitive pHAb label. In this context Weng et al. [12] found that pre-incubation of cells with nocodazole caused histidine tagged saporin to accumulate in larger vesicles in the presence of SA. These vesicles are possibly ECV since Baravalle et al. [35] reported that nocodazole caused dextran accumulation in large peripheral vesicles that were designated ECV. Baravalle et al. [35] also suggested that bafilomycin inhibits dextran transfer from early endosomes to late endocytic compartments whereas nocodazole affects transfer from ECV to late endosomes. Since we found that nocodazole had no effect on SA augmentation but that bafilomycin A1 abrogated SA augmentation, the release of SA and/or saporin may occur prior to the endosomal carrier vesicle to late endosome transfer stage of the endocytic pathway.

Holmes et al. [6] suggested that macropinocytosis might act as a mechanism by which SA is taken up by the cell. They proposed that SA is taken up non-specifically from the fluid phase by macropinocytosis and is then trafficked to an endocytic vesicle containing the antigen bound IT cargo taken up separately by RME. SA internalised in this way facilitates the escape of saporin or IT from the lumen of a disrupted endosomal or lysosomal vesicle at concentrations of IT or saporin that would normally prove less toxic in the absence of SA-mediated vesicle membrane disruption. We observed that EIPA, used as an inhibitor of macropinocytosis, had no significant effect on IT cytotoxicity alone but did abrogate the SA-mediated augmentation of all four ITs tested and non-targeted unconjugated saporin in both Daudi and HSB-2 cells. Confocal microscopy revealed that 4KB128-pHAb appeared to be less localised in Daudi cells treated with EIPA compared to control cells but the distribution of HB2-pHAb in EIPA treated HSB-2 cells was comparable to that seen in control cells. Despite the widespread use of EIPA as an inhibitor of macropinocytosis [21,22] numerous effects on other cellular processes such as endocytosis and those involving actin [36] have been reported. Amiloride derivatives are routinely used as Na^+^/H^+^ exchange (NHE1) inhibitors. Work by Koivasulo et al. [23] indicated that NHE1 activity is regulated to attain a critical H^+^ concentration in the immediate vicinity of the PM that promotes actin polymerisation during macropinocytosis. Therefore, it is difficult to conclude whether the effects of EIPA are truly indicative of the involvement of macropinocytosis or are the result of a direct effect on actin polymerisation or other mechanistic endocytic processes.

Holmes et al. [6] postulated a dual effect for the SA augmentation mechanism, firstly a direct increase in endosomal to cytosol escape of saporin resulting in toxin-mediated caspase dependent apoptosis [37] and secondly lysosomal-mediated cell death pathways triggered after the release of cathepsins and other hydrolytic enzymes following disruption of the lysosome membrane by putative saponin/ saporin complexes [25,38]. Honeychurch et al. [39] demonstrated that ROS play a critical role in lysosome mediated non-apoptotic cell death for monoclonal antibodies (mAb) directed against type I/type II anti-CD20 and HLA DR. The multiple death pathways induced by saporin-S6 containing ITs including apoptosis activation, autophagy, necroptosis, oxidative stress and the inhibition of protein synthesis have all been discussed in the literature [40]. A global gene expression analysis of two human malignant B-cell lines revealed that an IT containing saporin-S6 upregulated eleven genes involved in the cellular response to oxidative stress and DNA damage [41] suggesting to these authors that saporin-S6 induced a transcriptional response in cells that was dependent on oxidative stress/DNA damage leading to signal transduction blockage, cell cycle arrest and apoptosis.

Our own study has demonstrated that tiron, a superoxide scavenger, reduced the toxicity of OKT10-SAP towards Daudi cells whilst also reducing the intracellular levels of ROS generated following treatment with this IT. However, tiron did not reduce the augmentative effect of SA for OKT10-SAP in Daudi cells even though intracellular ROS levels were decreased by this superoxide scavenger. Whilst BU12-SAP cytotoxicity was also reduced by tiron, the cytotoxicities of 4KB128-SAP and unconjugated saporin were unaffected. Our results indicate that ROS are probably not involved in the SA-mediated augmentation of IT cytotoxicity. Polito et al. [42] reported that ROS were involved in the cytotoxic effect exerted by an anti-CD20-saporin-S6 IT but not that of an anti-CD22–saporin-S6 IT. They proposed that the different behaviour of the two ITs indicated that the induction of ROS by IT was target antigen-dependent and not directly due to the cytotoxic action of the toxin moiety. Our results appear to support this hypothesis.

Whilst our results with the various pharmacological agents are a useful indicator of the potential cellular mechanisms involved in the SA augmentation of ITs, caution should be applied in their interpretation. Small molecule pharmacological agents can exert other unanticipated cellular effects. For example, Lange et al. [43] showed that chlorpromazine increased the mobility of intracellular cholesterol and promoted the transfer of late endosomal and lysosomal cholesterol to serum in Nieman Pick type C cells. Kozik et al. [44] demonstrated a connection between endosome acidification, cholesterol trafficking and clathrin coated vesicle formation and found that long term bafilomycin treatment inhibited CME. They proposed a model for cholesterol distribution and function in clathrin coated pit formation in the presence/absence of a V-ATPase inhibitor. Hirama et al. [45] found that membrane curvature induced by the proximity of anionic phospholipids could initiate endocytosis and that cholesterol influenced membrane curvature by modulating the space between anionic phospholipids. In this respect it was recently reported that augmentation of the saporin IT BU12-SAP by SA is cholesterol dependent [10]. It is conceivable that some or possibly all of the effective small molecule pharmacological agents used in our study invoke other unforeseen changes in membrane structure via different mechanisms which in turn affect the release of SA or saporin from the lumen of late endosomes into the cytosol. To overcome the limitations of small pharmacological agents we propose to conduct experiments using siRNA knockdown of specific genes involved in endocytosis and intracellular vesicular function to corroborate the findings described here and to further clarify the mechanism(s) behind SA augmentation of IT cytotoxicity.

In summary we have shown, using a range of small molecule pharmacological agents, that endocytic processes are involved in the augmentative action of SA for saporin ITs directed against a range of lineage restricted antigens expressed by malignant lymphoid cells. The effects of the various pharmacological agents on SA augmentation of IT cytotoxicity cannot be ascribed to changes in expression levels or antibody internalisation rates. We found that there was no single common effect on cell cycle distribution by all five pharmaceutical agents that reduce the augmentative effect of SA. Inhibition of CME, actin polymerisation and endolysosomal acidification blocked the augmentative effect of SA whereas nocodazole, a compound that affects transfer from ECV to late endosomes, had no effect. These results indicate that a pH of below 6 is required for SA triterpenoid saponins to exert their augmentive effect on saporin IT cytotoxicity and possibly that SA initially acts mechanistically in the lumen of early endosomes. In addition, we have shown that ROS do not appear to play a role on the augmentative effect of SA for the range of saporin-based ITs with different target molecule specificities studied here.

## 4. Materials and Methods

### 4.1. Materials

Chloroquine (C6628), chlorpromazine (C0982), ethylisopropylamiloride (EIPA) (A3085), RPMI 1640 (R0883), phenolphthalein-free RPMI 1640 (R7509), sodium pyruvate (S8636), glutamine (G7513), foetal calf serum (FCS) (F0926), Tiron (172553) and bisBenzimide Hoechst 33342 trihydrochloride (B2261) were obtained from Merck (Sigma-Aldrich Company Ltd., Gillingham, UK). Bafilomycin A1 (CA11038) was from the Cayman Chemical Company (Ann Arbor, MI, USA) and nocodazole (358240100 Acros Organics) was supplied by Fisher Scientific UK Ltd. (Loughborough, UK). Cytochalasin D (PHZ1063), the CellRox Green Flow cytometry assay kit (Invitrogen C10492), Carboxy H_2_DCFDA (Invitrogen C400), Propidium iodide (P3566) and Ribonuclease A (EN0531) were from Thermofisher Scientific (Loughborough, UK). Rabbit F(ab’)2 Anti-mouse IgG:FITC (STAR9B) was from Bio-Rad Antibodies (Kidlington, UK). The pHAb amine reactive dye (G9845) was from Promega (Southampton, UK).

### 4.2. Cell Lines

The Daudi human Burkitt Lymphoma cell line and the T-cell acute lymphoblastic leukaemia cell line HSB-2 were obtained from the European Collection of Cell Cultures (ECACC, Porton Down, Salisbury, UK). Both cell lines were authenticated using the Identifier Plus DNA profiling system (Applied Biosciences, Carlsbad, CA, USA). Cells from a working cell bank were routinely passaged, for no greater than four weeks, in RPMI 1640 medium containing 10% foetal calf serum (FCS) supplemented with 2 mM glutamine and 2 mM sodium pyruvate (referred to hereafter as R10) at 37 °C in 7% CO_2_ in a humidified environment.

### 4.3. Saponinum Album (SA)

SA, a mixture of saponins from Gypsophila plant species, was obtained as a commercial preparation from Merck (Darmstadt, Germany) and characterized as described by Weng et al. [46]. The chemical structures for two of the most abundant saponin species present in SA have been shown elsewhere [10].

### 4.4. Saporin

The SO6 form of saporin was extracted and purified from *Saponaria officinalis* L. (Soapwort) (Chiltern Seeds, Ulverston, Cumbria, UK) as described elsewhere [47].

### 4.5. ITs

HB2-SAPORIN, BU12-SAPORIN, 4KB128-SAPORIN and OKT10-SAPORIN were constructed by covalently coupling the appropriate monoclonal antibody to the SO6 form of saporin using the heterobifunctional cross linking reagent SPDP as described previously [48]. All ITs constructed in this way contained very similar antibody:toxin (A:B) ratios presented here as a percentage of total conjugate species present as follows: 1:1; ~70%; 1:2; ~20% and 1:3; ~10%. BU12-SAPORIN and OKT10-SAPORIN contained <1% free antibody whilst 4KB128-SAPORIN and HB2-SAPORIN contained >10% free antibody. The presence of free antibody in the latter two ITs did not influence their cytotoxicity in the XTT assay against target antigen positive target cells until the amount of excess antibody exceeded the molar equivalent of conjugate present in the mixture (unpublished data).

### 4.6. Cell Culture

All experiments were conducted in standard RPMI 1640 or in phenolphthalein-free RPMI 1640 for XTT assays. Both media contained 10% FCS and were supplemented with 2 mM glutamine and 2 mM sodium pyruvate (R10).

### 4.7. XTT Assays

Dose-response curves for the cytotoxicity of each saporin IT or non-targeted unconjugated saporin used individually or in the presence of SA were determined for quadruplicate cultures of target cells using a modified XTT assay first described by Scudiero et al. [49]. Cells (5 × 10^4^) were seeded into a total volume of 200 µL R10 with or without SA and IT in 96 well plates and incubated at 37 °C, 7% CO_2_ for 48 h. The plates were read on a BMG Fluostar plate reader using a spectral scan from 300–650 nm. Results were expressed as a percentage of control cells cultured in the medium or SA alone and the 50% inhibitory concentration (EC_50_) was determined from the intercept with the 50% level on the Y axis of the dose-response curve. The fold increase was calculated by dividing the EC_50_ value for IT without SA by the EC_50_ value with SA. All experiments were conducted independently on two or more occasions with similar results obtained each time. We have previously established that when it comes to measuring cellular cytotoxicity the XTT assay is approximately 2 logs less sensitive than ^3^H-leucine incorporation or ^3^H-thymidine incorporation, but the dose-response curves obtained using the three different methods do mirror each other but at a correspondingly lower level of sensitivity.

### 4.8. Flow Cytometry

Cell surface expression levels of CD7, CD19, CD22 and CD38 were determined by single colour flow cytometry using an Epics XL instrument (Beckman Coulter, Miami, FL, USA) running EXPO32 ADC analytical software or on a Cytoflex Flow Cytometer (Beckman Coulter) using CytExpert software (Version 2.1.0.92, Beckman Coulter Life Sciences, Indianapolis, IN, USA). Cells were stained with a saturating concentration of the relevant antibody followed by a 1:50 dilution of fluorescein isothiocyanate labelled rabbit anti-mouse IgG (STAR 9B).

### 4.9. Confocal Microscopy

Antibodies were conjugated to a pHAb amine reactive dye (Promega) according to the manufacturers’ instructions. pHAb conjugates were added to Daudi or HSB-2 cells at a final concentration of 10 µg/mL. Cells were incubated at 37 °C, 7% CO_2_ for 5 or 24 h. Hoechst 33342 was added to a final concentration of 5 µg/mL twenty minutes prior to the end of the incubation period. Cells were washed by centrifugation, re-suspended in 200 µL RPMI and added to Ibidi 8 well glass bottomed plates pre-prepared with Alcian blue. A 1% solution of Alcian blue in distilled water was added to the wells of the plate and left for 1 h. The solution was removed and the wells were washed with distilled water until the liquid was clear. Images were acquired using a Leica TCs-SP8 laser scanning confocal microscope on DMi8 inverted microscope stand with a HC PL APO CS2 63×/1.30 glycerol immersion objective zoom 2.25 and Leica LAS-X acquisition software at 37 °C. Excitation wavelengths of 405 nm and 561 nm were used for Hoechst 33342 and the pHAb conjugate respectively.

### 4.10. Cell Cycle Analysis

Cells were cultured in T25 flasks in phenolphthalein-free R10 supplemented with the relevant small molecule pharmacological agent for 48 h at 37 °C, 7% CO_2_. At the end of the incubation period cells were harvested, fixed in 70% ice cold ethanol, left at 4 °C for 1 h, washed twice by centrifugation at 2300 rpm for 5 min, and re-suspended in PBS containing 100 µg/mL ribonuclease A and 50 µg/mL propidium iodide. The samples were left at 4 °C for 1 h before analysis by flow cytometry on a Cytoflex Flow Cytometer (Beckman Coulter) using selective gating to exclude doublet cells.

### 4.11. Detection of Reactive Oxygen Species (ROS)

The CellRox Green Flow Cytometry Assay Kit (Invitrogen) was used to detect ROS. The cell permeable CellRox Green is essentially non-fluorescent when in a reduced state but exhibits a strong fluorescent signal upon oxidation providing a measure of ROS in live cells. Cells were suspended in phenolphthalein-free R10 containing 500 nM CellRox Green and incubated at 37 °C, 7% CO_2_ for 30 min in the dark. The cell samples were centrifuged and re-suspended in 200 µL phenolphthalein-free RPMI before being analysed using FITC settings on a Cytoflex flow cytometer. Cells treated with 200 µM tert-butyl hydroperoxide were used as a positive control according to the manufacturer’s instructions.

The dye 5-(and 6)-carboxy-2′, 7′-dichlorodihydrofluorescein diacetate (carboxy-H_2_DCFDA) was also used as a probe for ROS detection in live cell imaging studies. Daudi cells (1 × 10^6^) were incubated in 400 µL 10 µM carboxy-H_2_DCFDA for 30 min at 37 °C, 7% CO_2_ in the dark before being added to Ibidi glass bottomed 8 well microscopy dishes coated with Alcian blue as previously described above. Daudi cells treated with 0.1% hydrogen peroxide for 1 h were used as a positive control. Images were acquired immediately using a Leica TCs-SP8 laser scanning confocal microscope on DMi8 inverted microscope stand with a HC PL APO CS2 63×/1.30 glycerol immersion objective zoom 2.25 and Leica LAS-X acquisition software at 37 °C. An excitation wavelength of 488 nm was used.

### 4.12. Statistical Analyses

Results are presented as mean and standard deviations. The number of data points used to determine mean and standard deviations are given in the individual figure legends along with the number of independent experiments carried out. The Student’s *t* test was used to determine *p* values for the flow cytometry data and EC_50_ ratio data. The significance of differences between the dose-response curves for the XTT data were evaluated using two-way repeated measure ANOVA using Graph Pad Prism.

## Figures and Tables

**Table 1:** EC_50_ without SA (saponinum album)/EC_50_ with SA ratio for each IT (immunotoxin) in Daudi cells. Values presented are calculated as the EC_50_ value without SA/EC_50_ value with SA for each individual IT or for unconjugated saporin in the presence and absence of bafilomycin A1 (Baf A), chlorpromazine (CPZ), chloroquine (CQN), cytochalasin D (Cyt D), EIPA or nocodazole (Noc). Error bars represent standard deviation of the mean of three EC_50_ ratios.

Toxin	Control	Baf A	CPZ	CQN	Cyt D	EIPA	Noc
Saporin	1.5 × 10^3^ ± 320	1.6 ± 0.4	2.1 ± 0.7	1.6 ± 0.8	4.3 ± 0.9	2.24 ± 1.6	2.3 × 10^3^ ± 158
OKT10-SAP	240 ± 87	0.6 ± 0.4	14.3 ± 8.1	0.6 ± 0.4	1.3 × 10^3^ ± 89	1.2 ± 0.9	300 ± 189
HB2-SAP	251 ± 15	2.1 ± 0.8	2.9 ± 0.7	1 ± 0.2	327 ± 46	1.3 ± 0.02	1.3 × 10^3^ ± 941
BU12-SAP	4.8 × 10^4^ ± 2.9 × 10^3^	309 ± 80	31.4 ± 7.8	27.5 ± 17.8	6.9 × 10^3^ ± 3 × 10^3^	0.25 ± 0.08	5.7 × 10^4^ ± 4 × 10^4^
4KB128-SAP	625 ± 360	0.5 ± 0.04	1 ± 0.11	2.7 ± 0.2	6.5 ± 3.8	0.79 ± 0.2	852 ± 74

## Supplementary Materials

The following are available online at http://www.mdpi.com/2072-6651/11/2/127/s1, Figure S1: Dose-response curves obtained by the XTT assay for Daudi cells exposed to bafilomycin A1 (Baf A) (A), chlorpromazine (B), chloroquine (C), cytochalasin D (Cyt D) (D), EIPA (F) or nocodazole (E), Figure S2: Dose-response curves obtained by the XTT assay for HSB-2 cells exposed to bafilomycin A1 (Baf A) (A), chlorpromazine (B), chloroquine (C), cytochalasin D (Cyt D) (D), EIPA (F) or nocodazole (E), Figure S3: The effects of pharmacological agents on SA augmentation of OKT10-SAP cytotoxicity in HSB-2 cells, Figure S4: The effects of pharmacological agents on SA augmentation of OKT10-SAP cytotoxicity in Daudi cells, Figure S5: The effects of pharmacological agents on SA augmentation of BU12-SAP cytotoxicity in Daudi cells, Figure S6: The effects of pharmacological agents on SA augmentation of saporin cytotoxicity in Daudi cells, Figure S7: The effects of pharmacological agents on SA augmentation of HB2-SAP cytotoxicity in Daudi cells, Figure S8: The effects of pharmacological agents on SA augmentation of 4KB128-SAP cytotoxicity in HSB-2 cells, Figure S9: The effects of pharmacological agents on SA augmentation of HB2-SAP cytotoxicity in HSB-2 cells, Figure S10: The effects of pharmacological agents on SA augmentation of saporin cytotoxicity in HSB-2 cells, Figure S11: The effects of pharmacological agents on SA augmentation of BU12-SAP cytotoxicity in HSB-2 cells, Figure S12: The effects of tiron on SA augmentation of BU12-SAP or 4KB128-SAP in Daudi cells, Table S1: EC50 values for each individual IT or for unconjugated saporin for HSB-2 cells exposed to bafilomycin A1 (Baf A), chlorpromazine (CPZ), chloroquine (CQN), cytochalasin D (Cyt D), EIPA or nocodazole (Noc).
