# Supplementary material for: Augmentation of Saporin-Based Immunotoxins for Human Leukaemia and Lymphoma Cells by Triterpenoid Saponins: The Modifying Effects of Small Molecule Pharmacological Agents
Source: Toxins (Basel). 2019 Feb 20;11(2):127. doi: 10.3390/toxins11020127 (PMC6410249; doi:10.3390/toxins11020127)
Supplement: Supplementary file 1 [file toxins-11-00127-s001.pdf]

toxins

MDPI

Supplementary Materials: Augmentation of Saporin-Based Immunotoxins for Human Leukaemia and Lymphoma Cells by Triterpenoid Saponins: The Modifying Effects of Small Molecule Pharmacological Agents

Wendy S. Smith, David A. Johnston, Suzanne E. Holmes, Harrison J. Wensley, Sopsamorn U. Flavell
and David J. Flavell

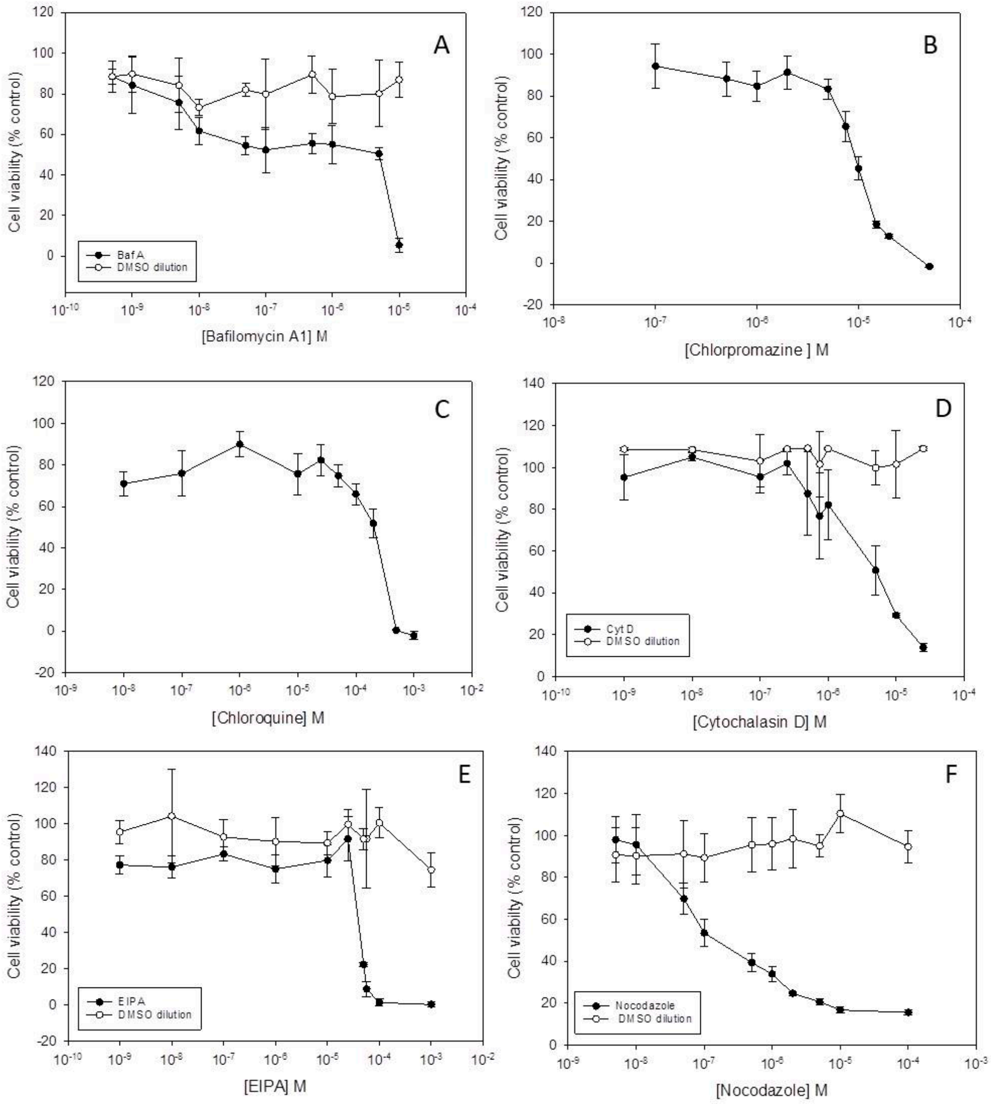

Figure S1. Dose response curves obtained by the XTT assay for Daudi cells exposed to bafilomycin A1 (Baf A) (**A**), chlorpromazine (**B**), chloroquine (**C**), cytochalasin D (Cyt D) (**D**), EIPA (**F**) or nocodazole (**E**). Samples were blank corrected and the absorbance at 470–650 nm was calculated for each well. Results are expressed as a percentage of control cells cultured in the relevant medium alone. Error bars represent the standard deviation either side of the mean for quadruplicate cultures.

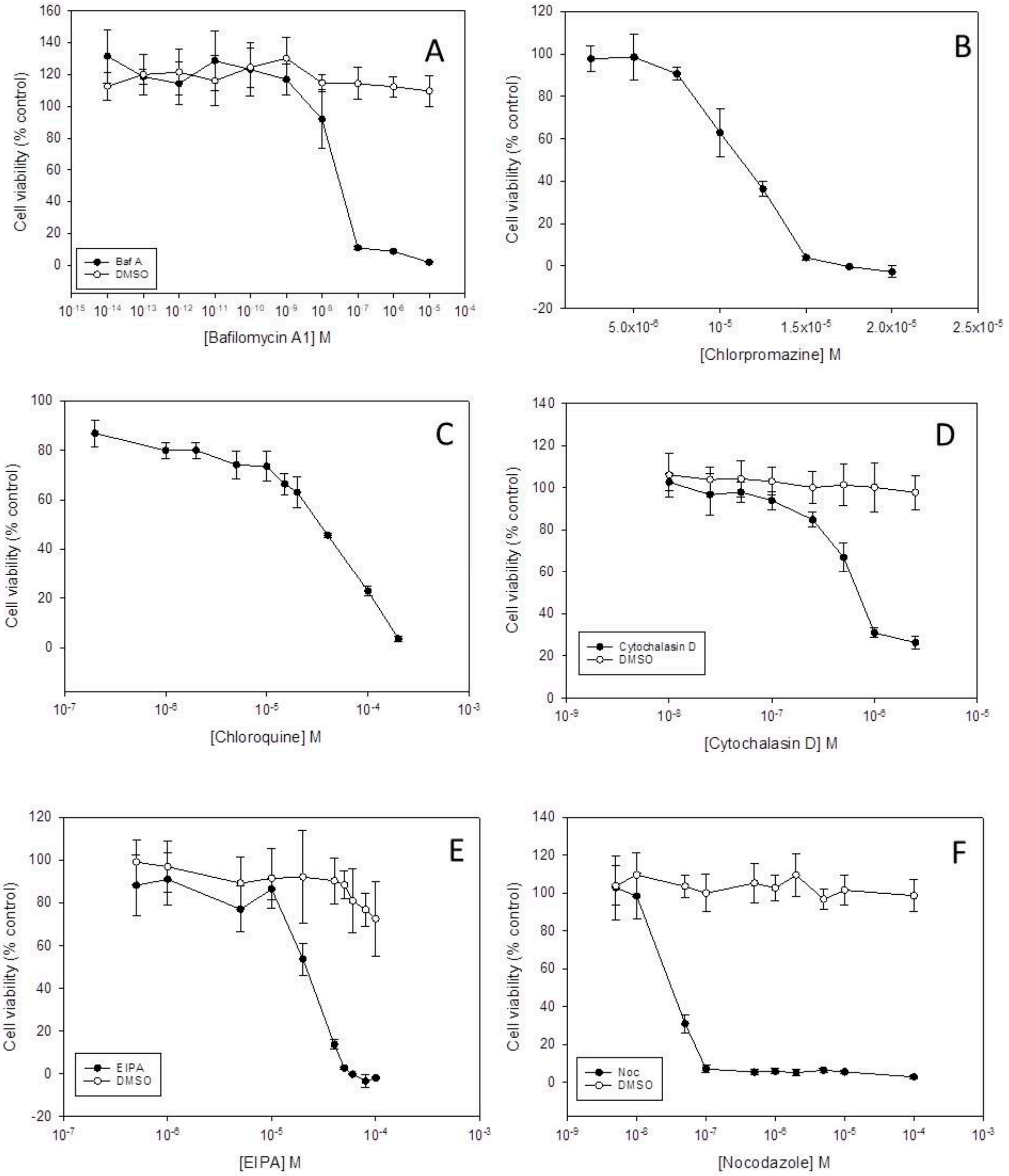

Figure S2. Dose response curves obtained by the XTT assay for HSB-2 cells exposed to bafilomycin A1 (Baf A) (**A**), chlorpromazine (**B**), chloroquine (**C**), cytochalasin D (Cyt D) (**D**), EIPA (**F**) or nocodazole (**E**). Samples were blank corrected and the absorbance at 470–650 nm was calculated for each well. Results are expressed as a percentage of control cells cultured in the relevant medium alone. Error bars represent the standard deviation either side of the mean for quadruplicate cultures.

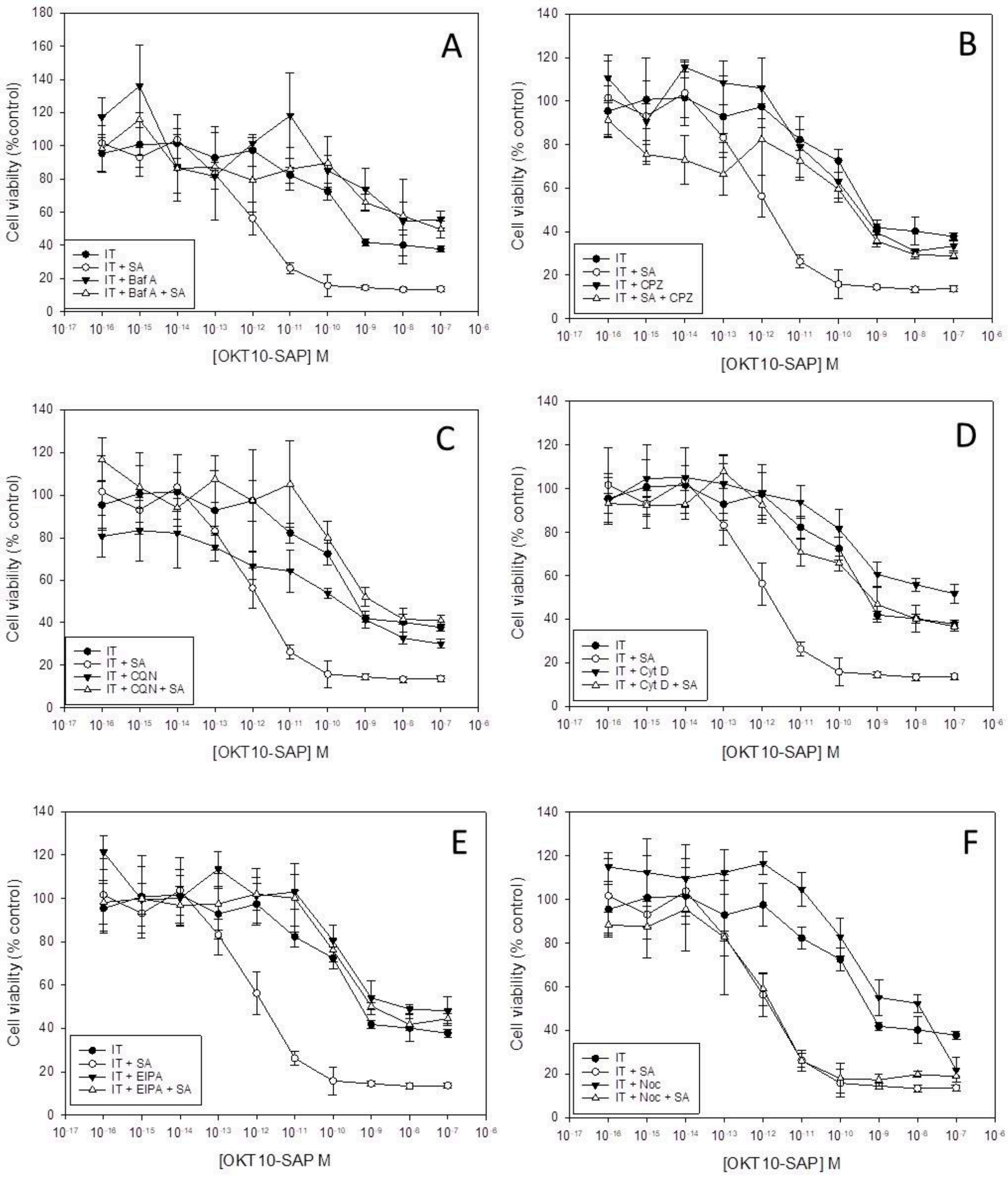

Figure S3. The effects of pharmacological agents on SA augmentation of OKT10-SAP cytotoxicity in HSB-2 cells. Dose response curves obtained using the XTT cytotoxicity assay for HSB-2 cells, in the absence (●) or presence (○) of 1 µg/mL SA, exposed to increasing concentrations of OKT10-SAP compared to cells incubated with bafilomycin A1 (Baf A) (**A**), chlorpromazine (CPZ) (**B**), chloroquine (CQN) (**C**), cytochalasin D (Cyt D) (**D**), EIPA (**E**) or nocodazole (Noc) (**F**) also exposed to increasing concentrations of OKT10-SAP in the absence (▼) or presence = (Δ) of 1 µg/mL SA. Samples were blank corrected and the absorbance at 470–650 nm was calculated for each well. Results are expressed as a percentage of control cells cultured in the relevant medium alone. Error bars represent the standard deviation either side of the mean for quadruplicate cultures and the data are representative of two or three independent experiments. Differences between the curves obtained for cells with IT plus SA without inhibitor and cells treated in the same way but with Baf A, Cpz, Cqn, Cyt D and EIPA were significant at the $p < 0.05$ level.

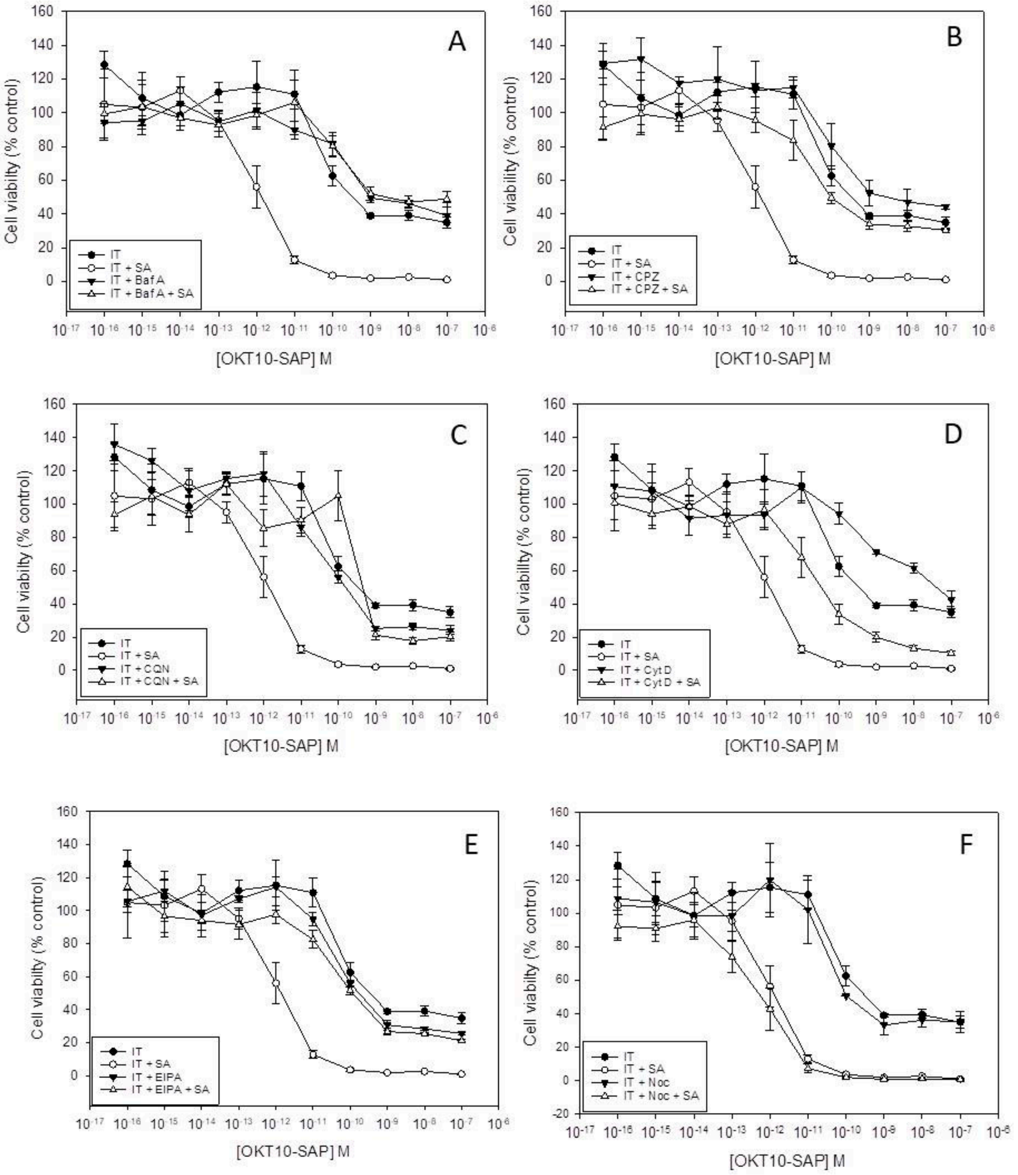

Figure S4. The effects of pharmacological agents on SA augmentation of OKT10-SAP cytotoxicity in Daudi cells. Dose response curves obtained by the XTT cytotoxicity assay for mock treated Daudi cells, in the absence (●) or presence (○) of 1 µg/mL SA, exposed to increasing concentrations of OKT10-SAP compared to cells incubated with bafilomycin A1 (Baf A) (**A**), chlorpromazine (CPZ) (**B**), chloroquine (CQN) (**C**), cytochalasin D (Cyt D) (**D**), EIPA (**E**) or nocodazole (Noc) (**F**) in the absence (▼) or presence (Δ) of 1 µg/mL SA. Samples were blank corrected and the absorbance at 470–650 nm was calculated for each well. Results are expressed as a percentage of control cells cultured in the relevant medium alone. Error bars represent the standard deviation either side of the mean for quadruplicate cultures and the data are representative of at least three independent experiments.

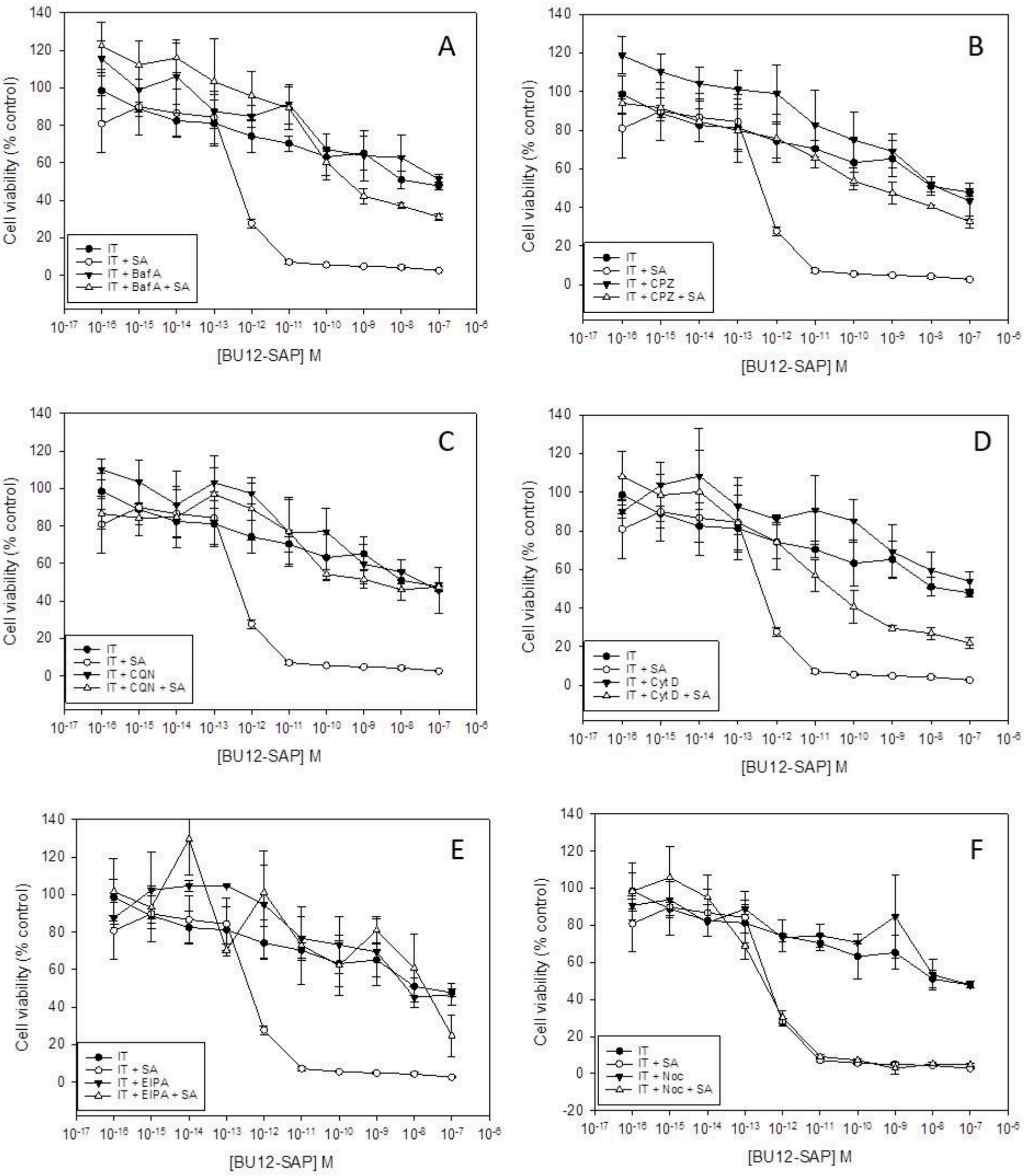

Figure S5. The effects of pharmacological agents on SA augmentation of BU12-SAP cytotoxicity in Daudi cells. Dose response curves obtained by the XTT cytotoxicity assay for mock treated Daudi cells, in the absence (●) or presence (○) of 1 µg/mL SA, exposed to increasing concentrations of BU12-SAP compared to cells incubated with bafilomycin A1 (Baf A) (**A**), chlorpromazine (CPZ) (**B**), chloroquine (CQN) (**C**), cytochalasin D (Cyt D) (**D**), EIPA (**E**) or nocodazole (Noc) (**F**) in the absence (▼) or presence (Δ) of 1 µg/mL SA. Samples were blank corrected and the absorbance at 470–650 nm was calculated for each well. Results are expressed as a percentage of control cells cultured in the relevant medium alone. Error bars represent the standard deviation either side of the mean for quadruplicate cultures and the data are representative of three independent experiments.

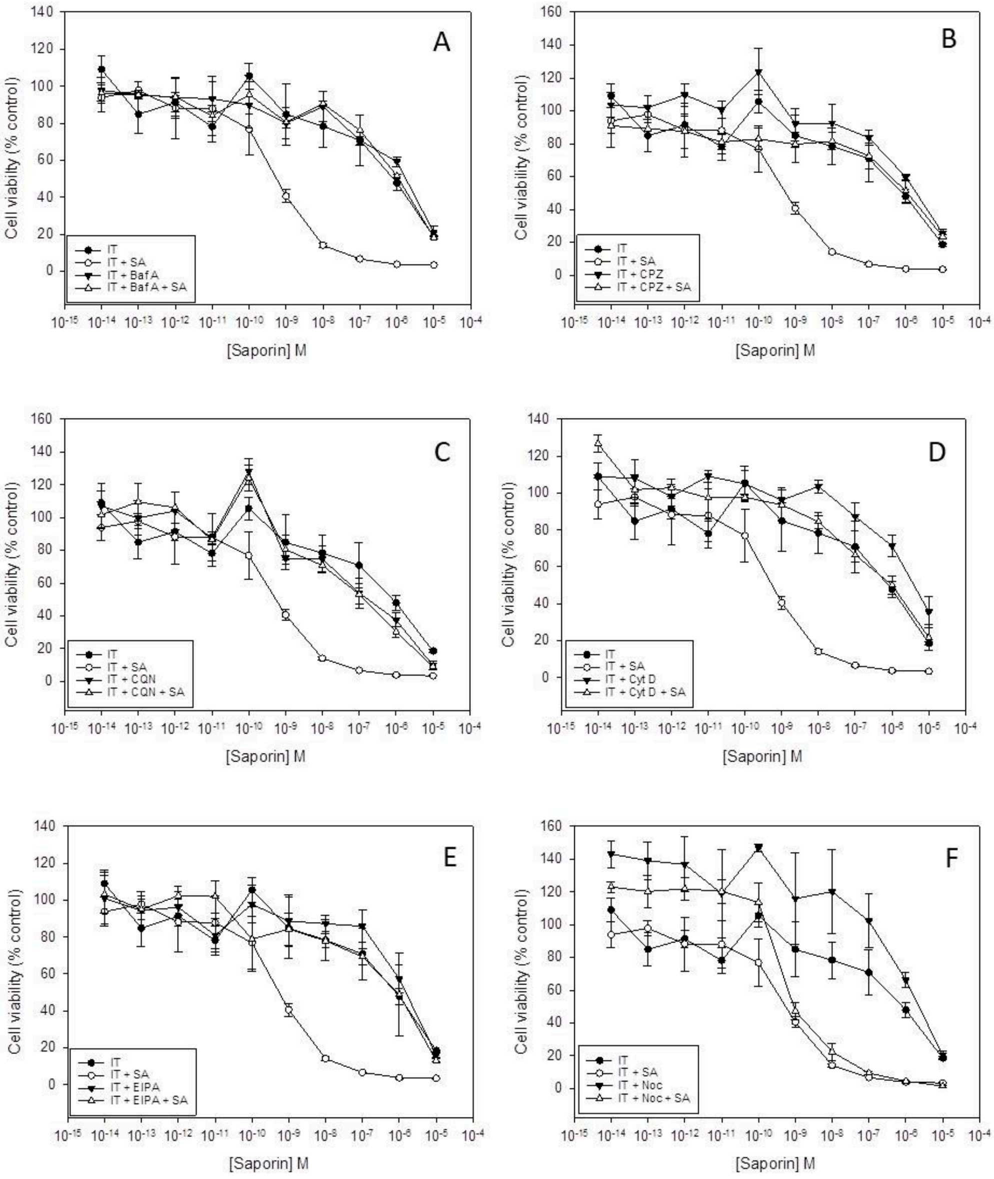

Figure S6. The effects of pharmacological agents on SA augmentation of saporin cytotoxicity in Daudi cells. Dose response curves obtained by the XTT cytotoxicity assay for mock treated Daudi cells, in the absence (●) or presence (○) of 1 µg/mL SA, exposed to increasing concentrations of Saporin compared to cells incubated with bafilomycin A1 (Baf A) (**A**), chlorpromazine (CPZ) (**B**), chloroquine (CQN) (**C**), cytochalasin D (Cyt D) (**D**), EIPA (**E**) or nocodazole (Noc) (**F**) in the absence (▼) or presence (Δ) of 1 µg/mL SA. Samples were blank corrected and the absorbance at 470–650 nm was calculated for each well. Results are expressed as a percentage of control cells cultured in the relevant medium alone. Error bars represent the standard deviation either side of the mean for quadruplicate cultures and the data are representative of three independent experiments.

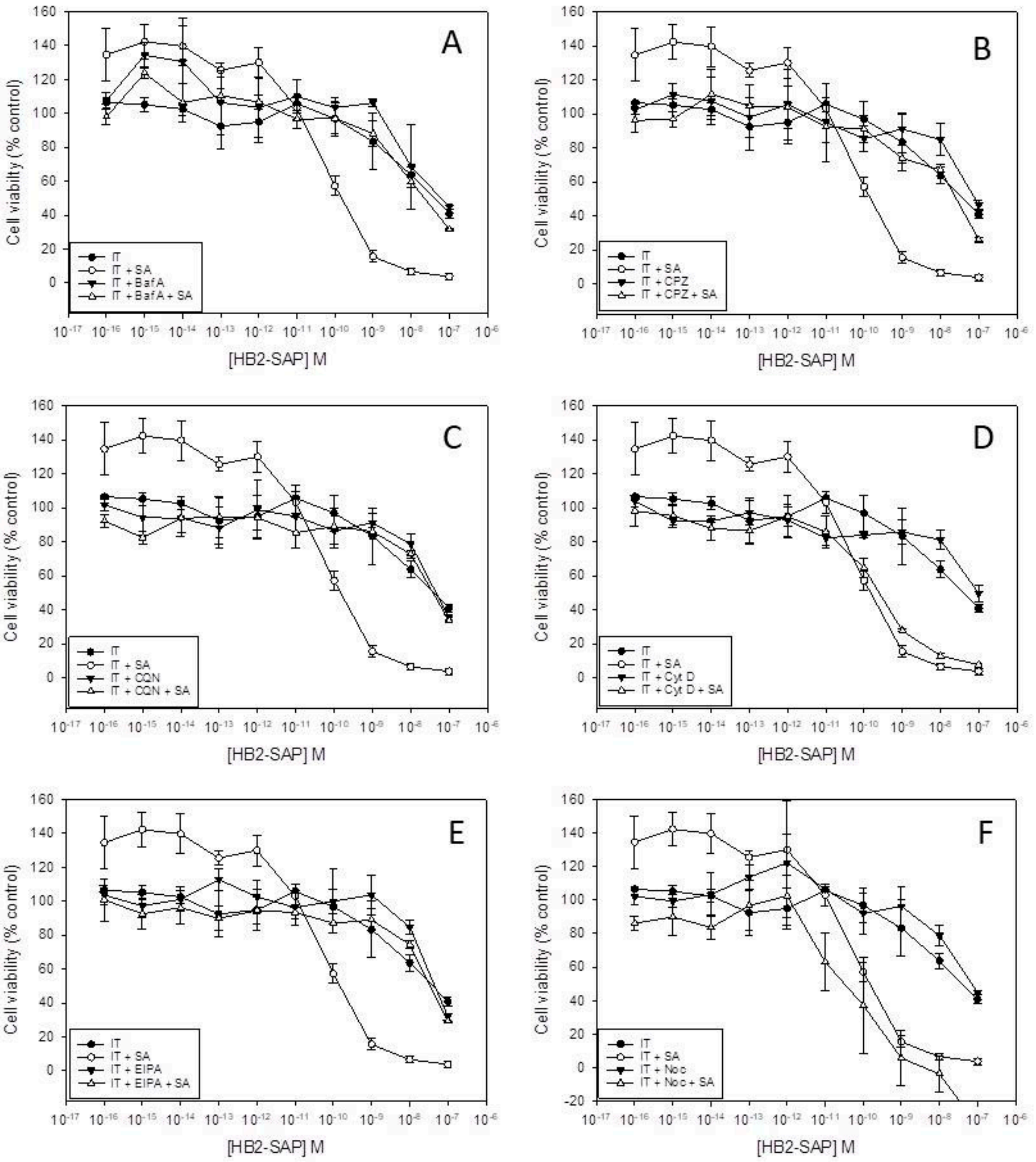

Figure S7. The effects of pharmacological agents on SA augmentation of HB2-SAP cytotoxicity in Daudi cells. Dose response curves obtained by the XTT cytotoxicity assay for mock treated Daudi cells, in the absence (●) or presence (○) of 1 μg/mL SA, exposed to increasing concentrations of HB2-SAP compared to cells incubated with bafilomycin A1 (Baf A) (**A**), chlorpromazine (CPZ) (**B**), chloroquine (CQN) (**C**), cytochalasin D (Cyt D) (**D**), EIPA (**E**) or nocodazole (Noc) (**F**) in the absence (▼) or presence (Δ) of 1 μg/mL SA. Samples were blank corrected and the absorbance at 470–650 nm was calculated for each well. Results are expressed as a percentage of control cells cultured in the relevant medium alone. Error bars represent the standard deviation either side of the mean for quadruplicate cultures and the data are representative of three independent experiments.

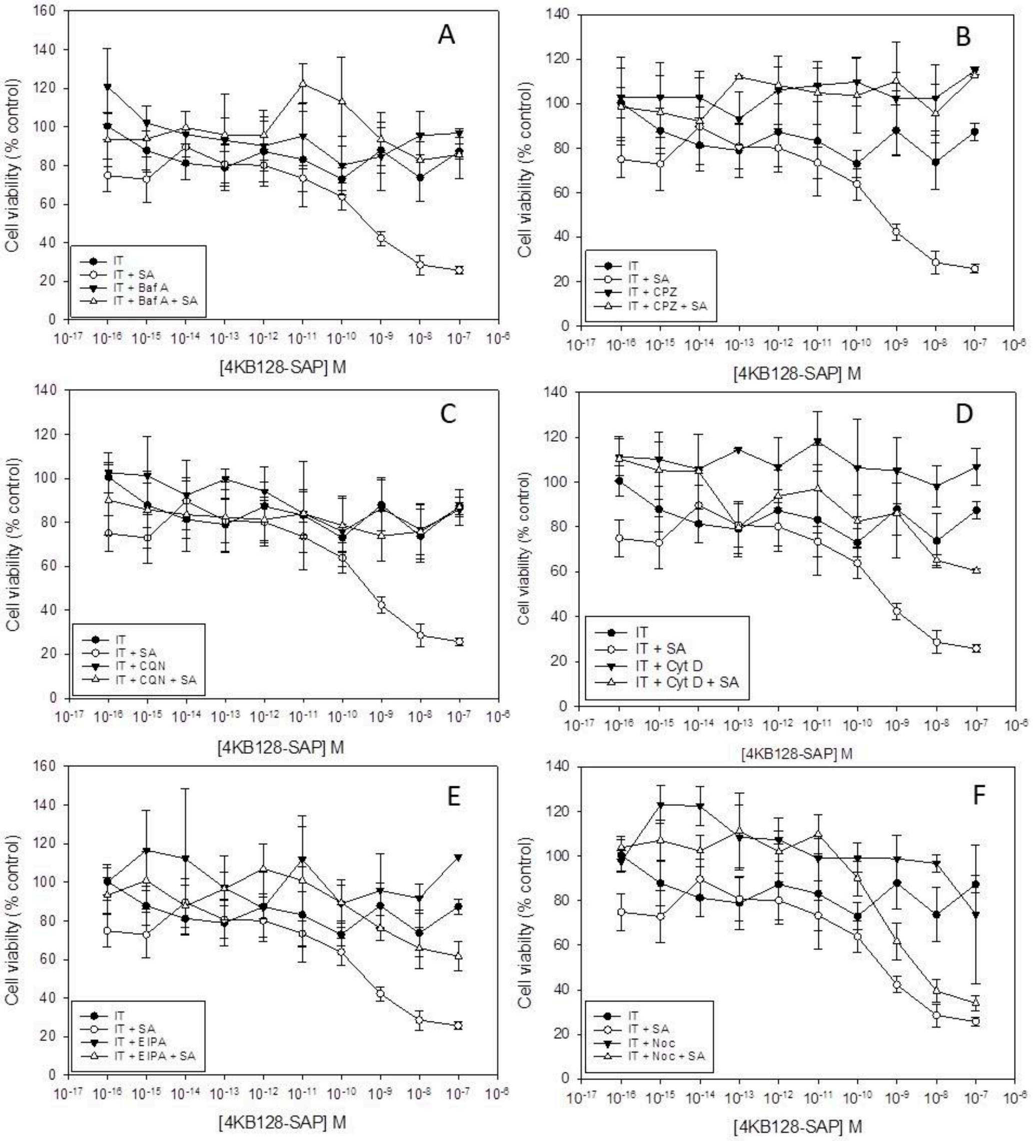

Figure S8. The effects of pharmacological agents on SA augmentation of 4KB128-SAP cytotoxicity in HSB-2 cells**.** Dose response curves obtained using the XTT cytotoxicity assay for mock treated HSB-2 cells, in the absence (●) or presence (○) of 1 µg/mL SA, exposed to increasing concentrations of 4KB128-SAP compared to cells incubated with bafilomycin A1 (Baf A) (**A**), chlorpromazine (CPZ) (**B**), chloroquine (CQN) (**C**), cytochalasin D (Cyt D) (**D**), EIPA (**E**) or nocodazole (Noc) (**F**) also in the absence (▼) or presence (Δ) of 1 µg/mL SA. Samples were blank corrected and the absorbance at 470–650 nm was calculated for each well. Results are expressed as a percentage of control cells cultured in the relevant medium alone. Error bars represent the standard deviation either side of the mean for quadruplicate cultures and the data are representative of two or three independent experiments.

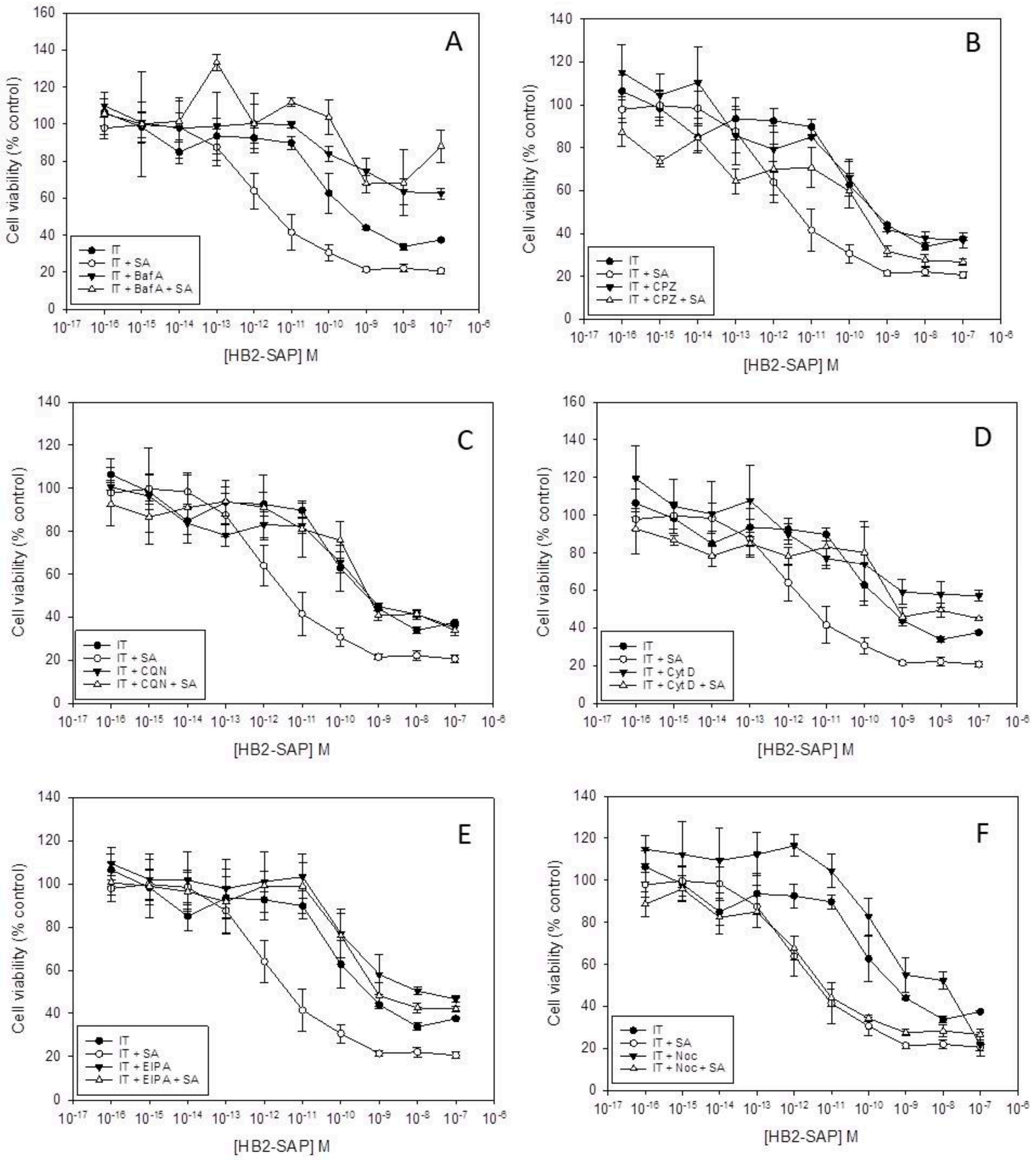

Figure S9. The effects of pharmacological agents on SA augmentation of HB2-SAP cytotoxicity in HSB-2 cells. Dose response curves obtained using the XTT cytotoxicity assay for mock treated HSB-2 cells, in the absence (●) or presence (○) of 1 µg/mL SA, exposed to increasing concentrations of HB2-SAP compared to cells incubated with bafilomycin A1 (Baf A) (**A**), chlorpromazine (CPZ) (**B**), chloroquine (CQN) (**C**), cytochalasin D (Cyt D) (**D**), EIPA (**E**) or nocodazole (Noc) (**F**) also in the absence (▼) or presence (Δ) of 1 µg/mL SA. Samples were blank corrected and the absorbance at 470–650 nm was calculated for each well. Results are expressed as a percentage of control cells cultured in the relevant medium alone. Error bars represent the standard deviation either side of the mean for quadruplicate cultures and the data are representative of two or three independent experiments.

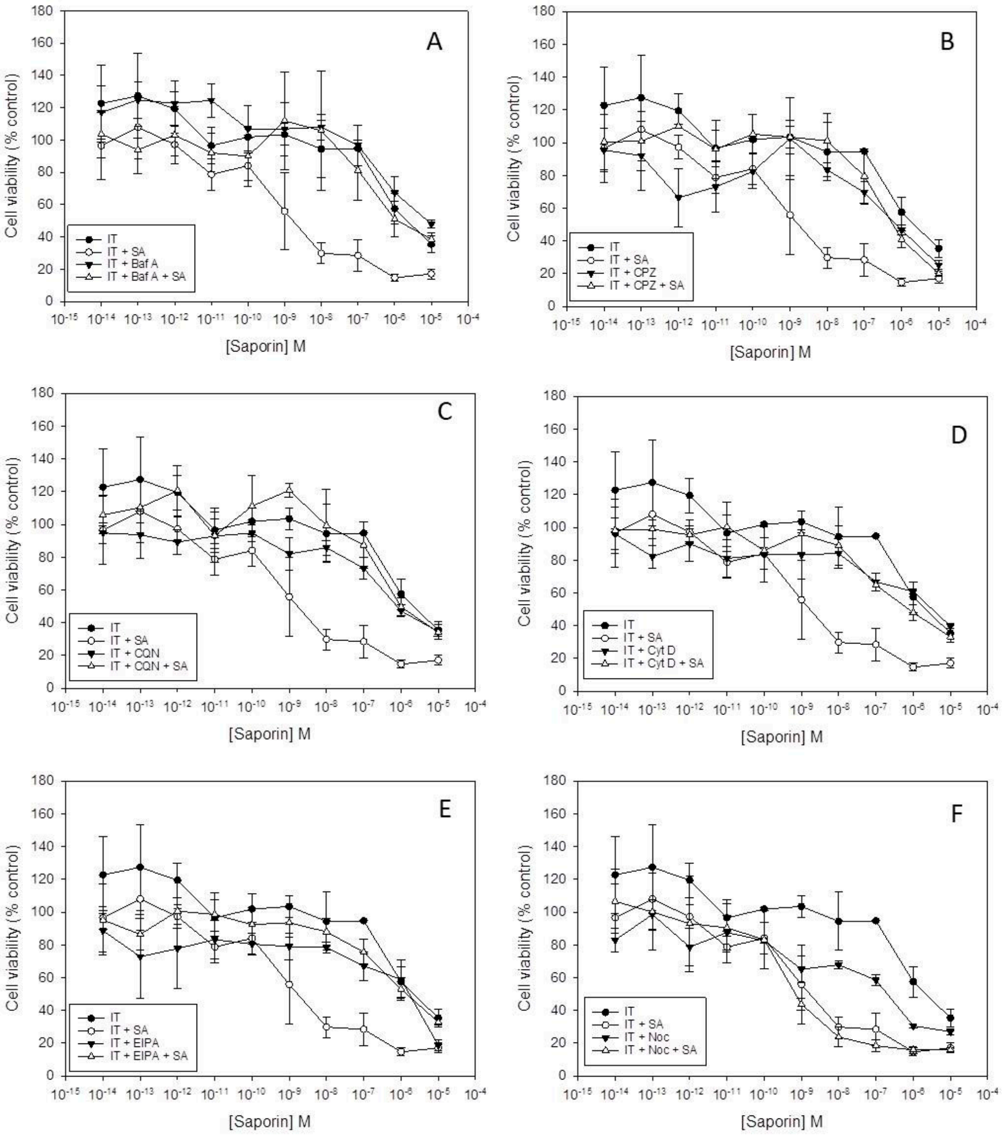

Figure S10. The effects of pharmacological agents on SA augmentation of saporin cytotoxicity in HSB-2 cells. Dose response curves obtained using the XTT cytotoxicity assay for mock treated HSB-2 cells, in the absence (●) or presence (○) of 1 μg/mL SA, exposed to increasing concentrations of Saporin compared to cells incubated with bafilomycin A1 (Baf A) (**A**), chlorpromazine (CPZ) (**B**), chloroquine (CQN) (**C**), cytochalasin D (Cyt D) (**D**), EIPA (**E**) or nocodazole (Noc) (**F**) also in the absence (▼) or presence (Δ) of 1 μg/mL SA. Samples were blank corrected and the absorbance at 470–650 nm was calculated for each well. Results are expressed as a percentage of control cells cultured in the relevant medium alone. Error bars represent the standard deviation either side of the mean for quadruplicate cultures and the data are representative of two or three independent experiments.

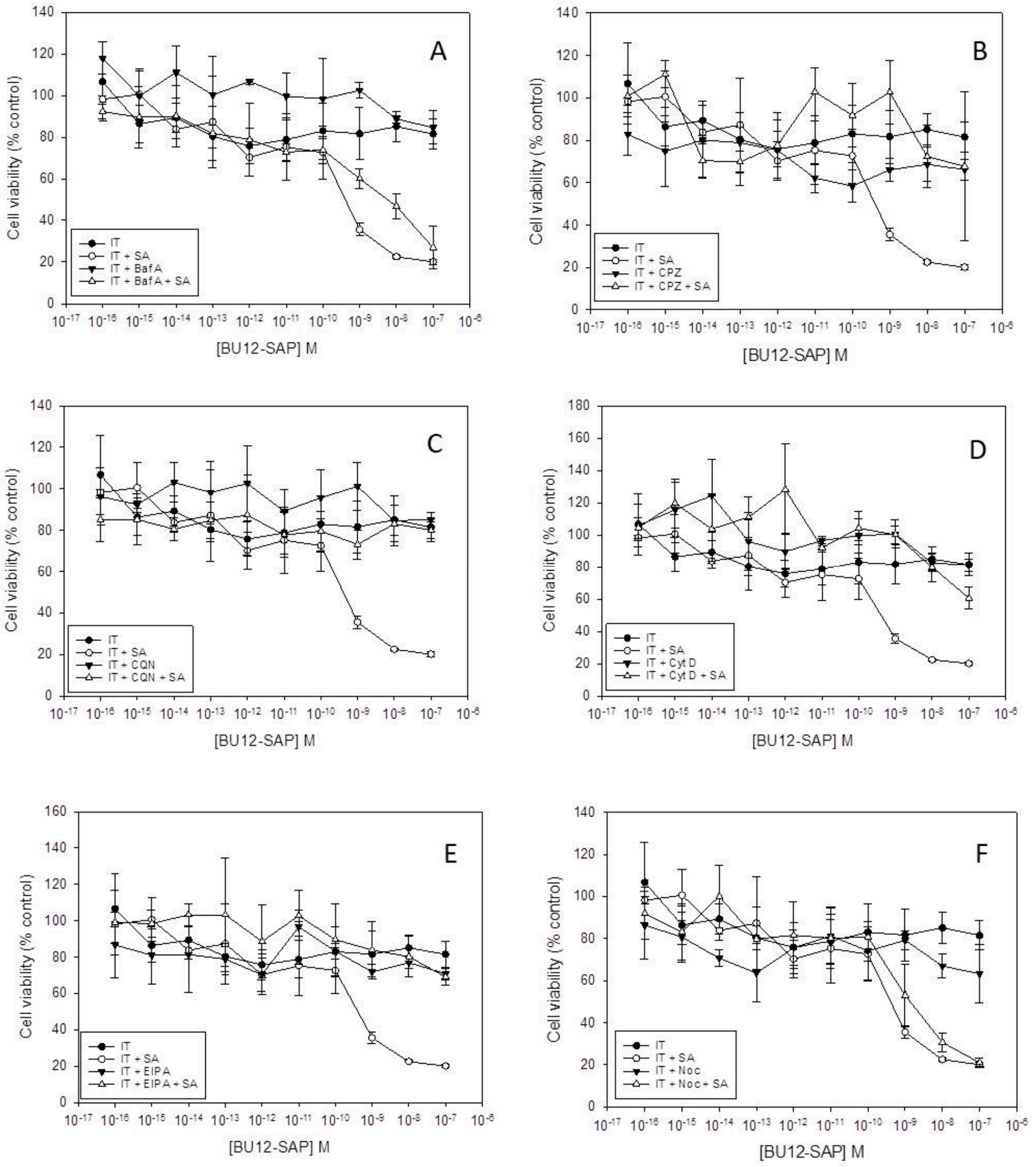

Figure S11. The effects of pharmacological agents on SA augmentation of BU12-SAP cytotoxicity in HSB-2 cells. Dose response curves obtained using the XTT cytotoxicity assay for mock treated HSB-2 cells, in the absence (●) or presence (○) of 1 μg/mL SA, exposed to increasing concentrations of BU12-SAP compared to cells incubated with bafilomycin A1 (Baf A) (**A**), chlorpromazine (CPZ) (**B**), chloroquine (CQN) (**C**), cytochalasin D (Cyt D) (**D**), EIPA (**E**) or nocodazole (Noc) (**F**) also in the absence (▼) or presence (Δ) of 1 μg/mL SA. Samples were blank corrected and the absorbance at 470–650 nm was calculated for each well. Results are expressed as a percentage of control cells cultured in the relevant medium alone. Error bars represent the standard deviation either side of the mean for quadruplicate cultures and the data are representative of two or three independent experiments.

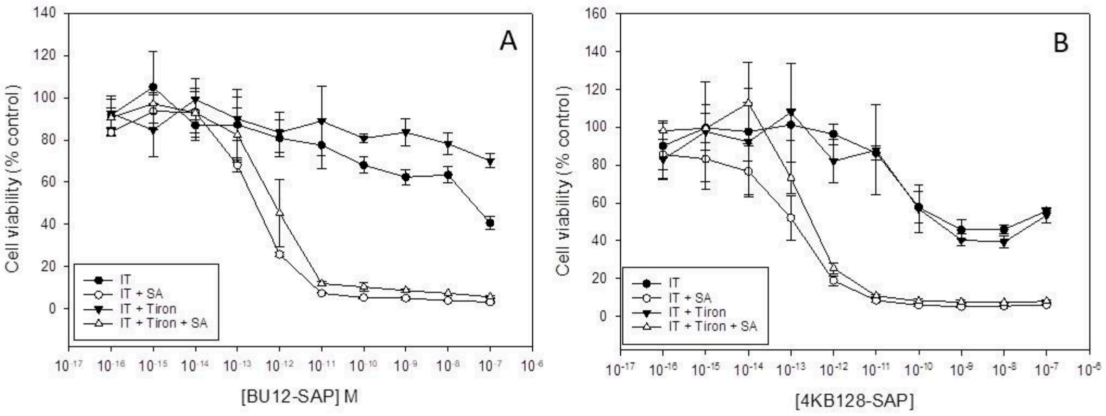

Figure S12. The effects of tiron on SA augmentation of BU12-SAP or 4KB128-SAP in Daudi cells. Dose-response curves obtained by the XTT assay for mock treated Daudi cells in the absence (●) or presence (○) of 1 µg/mL SA, exposed to increasing concentrations of OKT10-SAP (**A**) or saporin (**B**) compared to cells incubated with 500 µM tiron (**A**) in the absence (▼) or presence (Δ) of 1 µg/mL SA, following incubation in RPMI or 1mM tiron for 1 hour. Error bars represent the standard deviation either side of the mean for quadruplicate cultures and the data are representative of three independent experiments.

Table S1. EC_{50} values for each individual IT or for unconjugated saporin for HSB-2 cells exposed to bafilomycin A1 (Baf A), chlorpromazine (CPZ), chloroquine (CQN), cytochalasin D (Cyt D), EIPA or nocodazole (Noc). Values were determined from the intercept with the 50% level on the dose response curves.

HSB-2 Cells		EC_{50} Value				
		OKT10-SAP	HB2-SAP	BU12-SAP	4KB128-SAP	Saporin
Control	−SA	5.2×10^{-10}	4.2×10^{-10}	$>1 \times 10^{-7}$	$>1 \times 10^{-7}$	1.2×10^{-6}
	+SA	1.8×10^{-12}	4.0×10^{-12}	4.0×10^{-10}	4.0×10^{-10}	1.8×10^{-9}
Baf A	−SA	2×10^{-7}	$>1 \times 10^{-7}$	$>1 \times 10^{-7}$	$>1 \times 10^{-7}$	8.0×10^{-6}
	+SA	1×10^{-7}	$>1 \times 10^{-7}$	5.0×10^{-9}	$>1 \times 10^{-7}$	1.0×10^{-6}
CPZ	−SA	3.2×10^{-10}	5.0×10^{-10}	$>1 \times 10^{-7}$	$>1 \times 10^{-7}$	7.0×10^{-7}
	+SA	2.4×10^{-10}	2.0×10^{-10}	$>1 \times 10^{-7}$	$>1 \times 10^{-7}$	6.0×10^{-7}
CQN	−SA	2×10^{-10}	5.0×10^{-10}	$>1 \times 10^{-7}$	$>1 \times 10^{-7}$	7.5×10^{-7}
	+SA	1.6×10^{-9}	5.0×10^{-10}	$>1 \times 10^{-7}$	$>1 \times 10^{-7}$	1.0×10^{-6}
Cyt D	−SA	1×10^{-7}	1.0×10^{-8}	$>1 \times 10^{-7}$	$>1 \times 10^{-7}$	3.0×10^{-6}
	+SA	6.5×10^{-10}	2.5×10^{-9}	$>1 \times 10^{-7}$	$>1 \times 10^{-7}$	7.0×10^{-7}
EIPA	−SA	5.5×10^{-9}	1.0×10^{-8}	$>1 \times 10^{-7}$	$>1 \times 10^{-7}$	1.6×10^{-6}
	+SA	1×10^{-9}	9.0×10^{-10}	$>1 \times 10^{-7}$	$>1 \times 10^{-7}$	1.4×10^{-6}
Noc	−SA	2×10^{-9}	2.5×10^{-9}	$>1 \times 10^{-7}$	$>1 \times 10^{-7}$	2.0×10^{-7}
	+SA	2×10^{-12}	5.5×10^{-12}	1.5×10^{-9}	3.2×10^{-9}	7.0×10^{-10}
